# Review of cooling techniques used to enhance the efficiency of photovoltaic power systems

**DOI:** 10.1007/s11356-022-18719-9

**Published:** 2022-01-25

**Authors:** Mohamed Sharaf, Mohamed S. Yousef, Ahmed S. Huzayyin

**Affiliations:** Mechanical Engineering Department, Benha Faculty of Engineering, Benha University, Benha, Egypt

**Keywords:** Photovoltaic, Enhancement, Thermal management, Cooling, PCM, Porous metal

## Abstract

Photovoltaic (PV) panels are one of the most important solar energy sources used to convert the sun’s radiation falling on them into electrical power directly. Many factors affect the functioning of photovoltaic panels, including external factors and internal factors. External factors such as wind speed, incident radiation rate, ambient temperature, and dust accumulation on the PV cannot be controlled. The internal factors can be controlled, such as PV surface temperature. Some of the radiation falling on the surface of the PV cell turns into electricity, while the remainder of incident radiation is absorbed inside the PV cell. This, in turn, elevates its surface temperature. Undesirably, the higher panel temperature, the lower conversion performance, and lesser reliability over the long term occur. Hence, many cooling systems have been designed and investigated, aiming to effectively avoid the excessive temperature rise and enhance their efficiency. Many cooling methods are used to cool solar cells, such as passive cooling, active cooling, cooling with phase change materials (PCMs), and cooling with PCM with other additives such as nanoparticles or porous metal. In this work, the common methods utilized for cooling PV panels are reviewed and analyzed, focusing on the last methods, and summarizing all the researches that dealt with cooling PV solar cells with PCM and porous structures.

## Introduction

Excessive consumption of traditional fossil-based energies at a disturbing rate resulted in an energy crisis and was accountable for accompanying environmental concerns such as global warming, greenhouse harmful gas emissions, and ozone depilation that threaten the security of human beings and the future of the world (Abdulmunem et al., 2020). Therefore, greater demands for clean and renewable energy technologies are raised to reduce our dependency on fossil-based energies and abate its subsequent adverse environmental concerns. Solar energy is the best widespread and broadly prevalent accepted renewable energy source due to its cleanliness, simple accessibility, sustainability, and unlimited potential (Xu et al., 2021). The sun is the most powerful renewable energy source, as natural sunlight can heat and light houses and other buildings. In addition to that, solar energy is used in generating electricity, water heating, and a variety of other industrial processes. The methods used to harvest solar energy are constantly evolving, and their efficiency increases with the development of technology, including water heating tubes on rooftops, solar cells, and mirrors (Sharaf et al., 2021; Yousef et al., 2016). Most of the renewable energy produced on Earth is attributed to solar radiation and secondary energy sources, such as wind energy, wave energy, hydropower, and biomass (Abo-Elfadl et al., 2020a; Hassan and Yousef, 2021). It is worth noting that only a small fraction of the solar energy that reaches the earth is harvested. Once solar energy is converted into electrical energy, only human controls its use. Among the solar energy applications are the heating and cooling systems in architectural designs that depend on the exploitation of solar energy, potable water from distillation and disinfection processes, the exploitation of daylight, water heating, solar cooking, and high temperatures for industrial purposes.

### Different PV technologies

Photovoltaic (PV) systems range from small, rooftop-mounted, or building-integrated systems with capacities from a few to several tens of kilowatts, to large utility-scale power stations of hundreds of megawatts. Nowadays, most PV systems are grid-connected, while stand-alone systems only account for a small portion of the market. We will now show the most important applications of photovoltaic panels.

### Rooftop and building integrated systems

Photovoltaic arrays are often associated with buildings: either integrated into them, mounted on them, or mounted nearby on the ground. Building-integrated photovoltaics (BIPVs) are increasingly incorporated into the roof or walls of new domestic and industrial buildings as a principal or ancillary source of electrical power (Darby, 2018). Typically, residential rooftop systems have small capacities of around 5–10 kW, while commercial rooftop systems often amount to several hundreds of kilowatts. Although rooftop systems are much smaller than ground-mounted utility-scale power plants, they account for most of the worldwide installed capacity (Association, 2014).

### Photovoltaic thermal hybrid solar collector

Photovoltaic thermal (PVT) hybrid solar collectors are systems that convert solar radiation into thermal and electrical energy. These systems combine a solar PV cell, which converts sunlight into electricity, with a solar thermal collector, which captures the remaining energy and removes waste heat from the PV module. The capture of both electricity and heat allow these devices to have higher exergy and thus be more overall energy efficient than solar PV or solar thermal alone (Pathak et al., 2014).

### Power stations

Many utility-scale solar farms have been constructed all over the world. In 2011, the 579-megawatt (MW_AC_) Solar Star project was proposed, to be followed by the Desert Sunlight Solar Farm and the Topaz Solar Farm in the future, both with a capacity of 550 MW_AC_, to be constructed by US company First Solar, using CdTe modules, a thin-film PV technology. All three power stations will be located in the Californian desert. When the Solar Star project was completed in 2015, it was the world’s largest photovoltaic power station at the time (Wesoff, 2015).

### In transport

PV has traditionally been used for electric power in space. PV is rarely used to provide motive power in transport applications, but it can provide auxiliary power in boats and cars. Some automobiles are fitted with solar-powered air conditioning. A self-contained solar vehicle would have limited power and utility, but a solar-charged electric vehicle allows use of solar power for transportation. Solar-powered cars, boats, and airplanes have been demonstrated, with the most practical and likely of these being solar cars (Sukumaran and Sudhakar, 2017).

### Telecommunication and signaling

Solar PV power is ideally suited for telecommunication applications such as local telephone exchange, radio and TV broadcasting, microwave, and other forms of electronic communication links. This is because, in most telecommunication application, storage batteries are already in use and the electrical system is basically DC. In hilly and mountainous terrain, radio and TV signals may not reach as they get blocked or reflected back due to undulating terrain. At these locations, low-power transmitters (LPT) are installed to receive and retransmit the signal for local population (Tech, n.d.).

### 4G solar cell technology

Over the years, this technology has evolved from the first generation to the fourth generation, which we refer to as 4G solar cell technology. As such, since the discovery of the first photovoltaic effect in 1839 by Alexandre Becquerel, the use of solar energy has been a goal in the scientific world. Photovoltaic (solar cells) are basically grouped into four generations and this is based on the materials used: these are first-generation, second-generation, third-generation, and fourth-generation cells (Tala-ighil, 2015).

First-generation cells are the earliest photovoltaic cells produced using silicon in 1954 which had an efficiency of 6%. They are currently the most efficient solar cells for use in our homes, accounting for above 80% of all the solar panels sold globally. As the most abundant element on earth, second only to oxygen, it is therefore readily available for use as a semiconducting material suitable for PV applications (Aghenta and Iqbal, 2019). The second-generation solar cells are also referred to as thin-film solar cells. They are comprised of successive thin layers of solar cells deposited onto a large, cheap substrate such as metal and glass. The third-generation solar cell technology came into existence due to the high costs of the first generation, toxicity, and limited availability of solar cells for the second-generation technology. The third-generation solar cell technology make solar cells from a variety of materials apart from silicon. It uses silicon wires, nanomaterials, organic dyes, and conductive plastics, all in an attempt to devise a more efficient and readily available technology (Singh et al., 2021).

The fourth-generation solar cell technology is also referred to as the 4G solar cell technology. This technology makes use of the combination of inorganic and organic materials, as a means to boost the efficiency and cost-effectiveness of solar cells. The 4G solar cells are engineered at solar scale and are characterized by the flexibility of conducting polymer films (the organic materials), and the stable nanostructures (inorganic materials). In the 4G solar cells, the commonly used substrate is transparent tin doped indium oxide; however, new alternatives have made use of graphene, metal nanowires, and metal grid structures (Singh et al., 2021). The essence of the nanomaterials in these solar cells enables large volume of surrounding the nanomaterial to be filled using a conductor, such as a polymer. The main advantage of 4G solar cell over the other technologies is that the combination of organic and inorganic substrates improves the harvesting of solar energy, thereby ensuring better efficiency while also maintaining meaningful cost savings (Tala-ighil, 2015).

## Working principle of photovoltaic cell and temperature effect on its output power

Photovoltaic (PV) is one of the most established solar energy conversion technologies, which converts solar energy directly into electricity with unrestricted potential, noiseless operation, and little necessity for maintenance. The PV cell is basically a diode of the junction p-n. PV solar cells are a form of photoelectric cell, defined as a device whose electrical characteristics—such as voltage, current, or resistance—vary when exposed to light. Individual solar cells can be combined to form modules commonly known as solar panels. The common single-junction silicon solar cell can produce a maximum open-circuit voltage of approximately 0.5 to 0.6 V. By itself, this is not much—but remember, these solar cells are tiny. When combined into a large solar panel, considerable amounts of renewable energy can be generated (Krishan and Suhag, 2019).

**Fig. 1:**
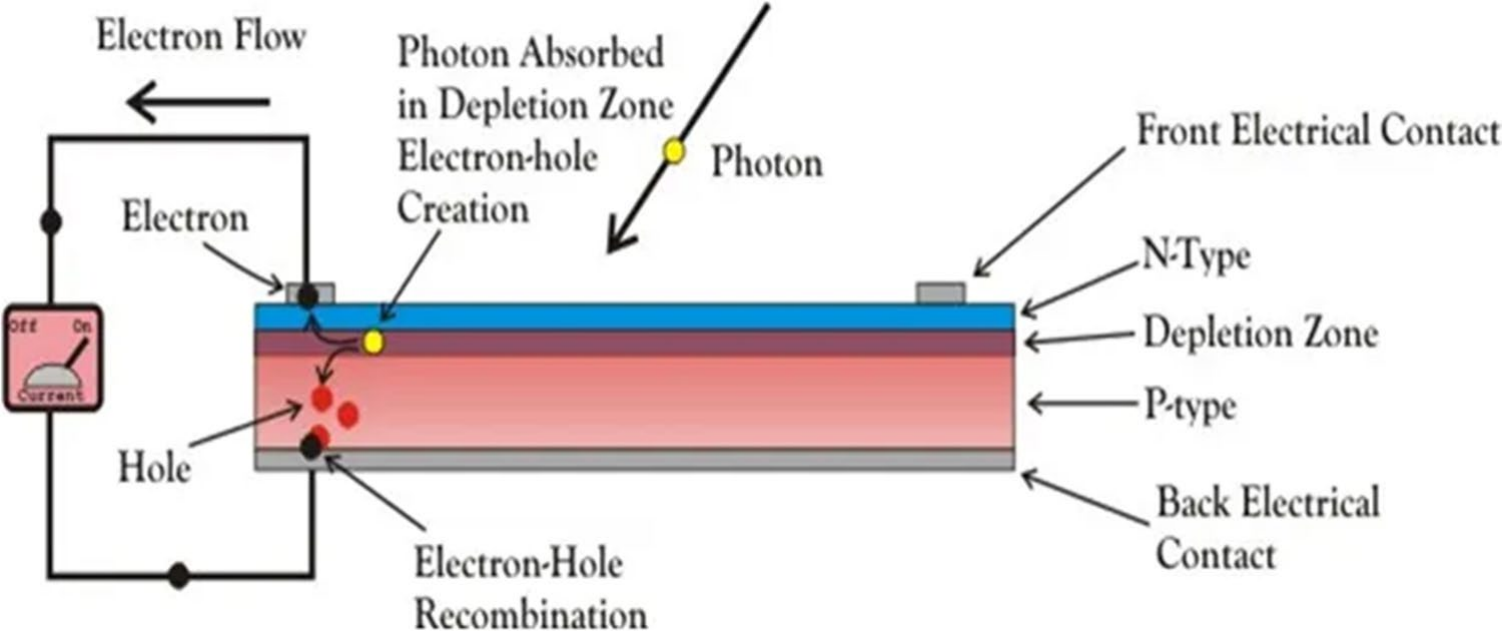
The working principle of (PV) (“https://www.electrical4u.com/,” n.d.)

When light reaches the p–n junction, the light photons can easily enter the junction through a very thin p-type layer. The light energy, in the form of photons, supplies sufficient energy to the junction to create several electron–hole pairs. The incident light breaks the thermal equilibrium condition of the junction. The free electrons in the depletion region can quickly come to the n-type side of the junction. Similarly, the holes in the depletion can quickly come to the p-type side of the junction (Jordehi, 2016), as illustrated in Fig. 1. Due to the motion of the electrons from the p-type side to the n-type side and vice versa, the voltage is produced, and if we connect a load through the junction, the current will flow through the circuit.

**Fig. 2:**
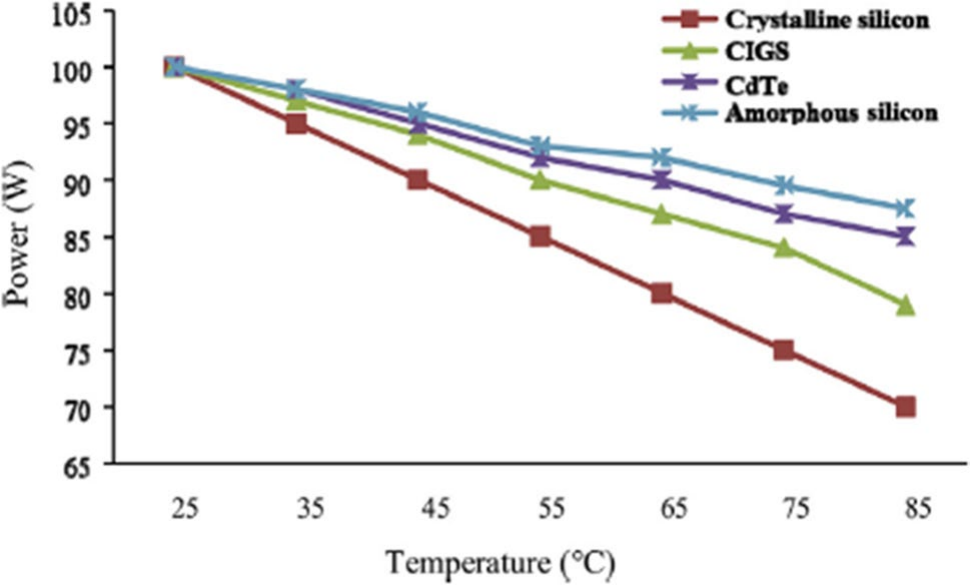
Influence of temperature on obtained power from PVs with various materials (Hasanuzzaman et al., 2016)

Due to the increase in surface temperature of the PV cell, the circuit resistance increases, and this limits the velocity of the electron, which directly affects the open-circuit voltage besides badly influencing the cell material. The voltage drop-in/temperature rise unit, called temperature coefficient, describes the temperature reliance of the particular material used for PV cell performance analysis and indicates the strong dependency of PV power output on the surface temperature of the PV. Manufacturers of PV modules usually provide the value of $\beta$_T_ in their product brochures. Figure 2 shows the effect of the temperature increase on the power output of the PV cell for various materials. The voltage drop is about 0.12 V, and thus the temperature coefficient is 0.12 V/°C for each 1 °C increase of cell temperature in the polycrystalline PV cell. Moreover, the STC performance and negative temperature coefficients of various kinds of solar cells are illustrated in Table 1 (Hasanuzzaman et al., 2016).

**Table 1:** Performance of different kinds of PV cells and their temperature coefficient (Hasanuzzaman et al., 2016)

Type	Temperature coefficient	STC performance (%)
Mono crystalline–Si (m-Si)	− 0.4 to − 0.5	12.5–15
Poly crystalline–Si (p-Si)	− 0.4 to − 0.5	11–14
Amorphous–Si (a-Si)	− 0.35 to − 0.38	11–13
CIGS	− 0.32 to − 0.36	1013
CdTe	− 0.25	9–12

In the present work, an extensive review of the numerous cooling methods employed to maintain the operating temperature of modules closer to the specified limit to enhance the PV modules performance is presented and for which an enormous number of research articles are referred. The cooling methods used are described under four broad categories: passive cooling techniques, active cooling techniques, PCM cooling, and PCM with additives. Many studies made a general review of the methods of cooling PV solar cells, especially the first three methods. Therefore, the current research is characterized by dealing with different cooling techniques, focusing on the fourth method, and summarizing all the researches that dealt with cooling PV solar cells with PCM and porous metal.

## Cooling techniques

Some of the radiation incidents on the PV solar cells are reflected from the PV module. Another percentage of the incident radiation is stored in the PV module as heat; this amount of heat causes raising in the surface temperature of the PV module. The ratio between the PV module’s electrical power and the solar radiation incident on the PV module is called the electrical efficiency of the PV module.

1 $${\upeta }_{\mathrm{PV module}}=\frac{\mathrm{The electrical power produced from the PV module}}{\mathrm{Solar radiation incident on the PV module}}$$

**Fig. 3:**
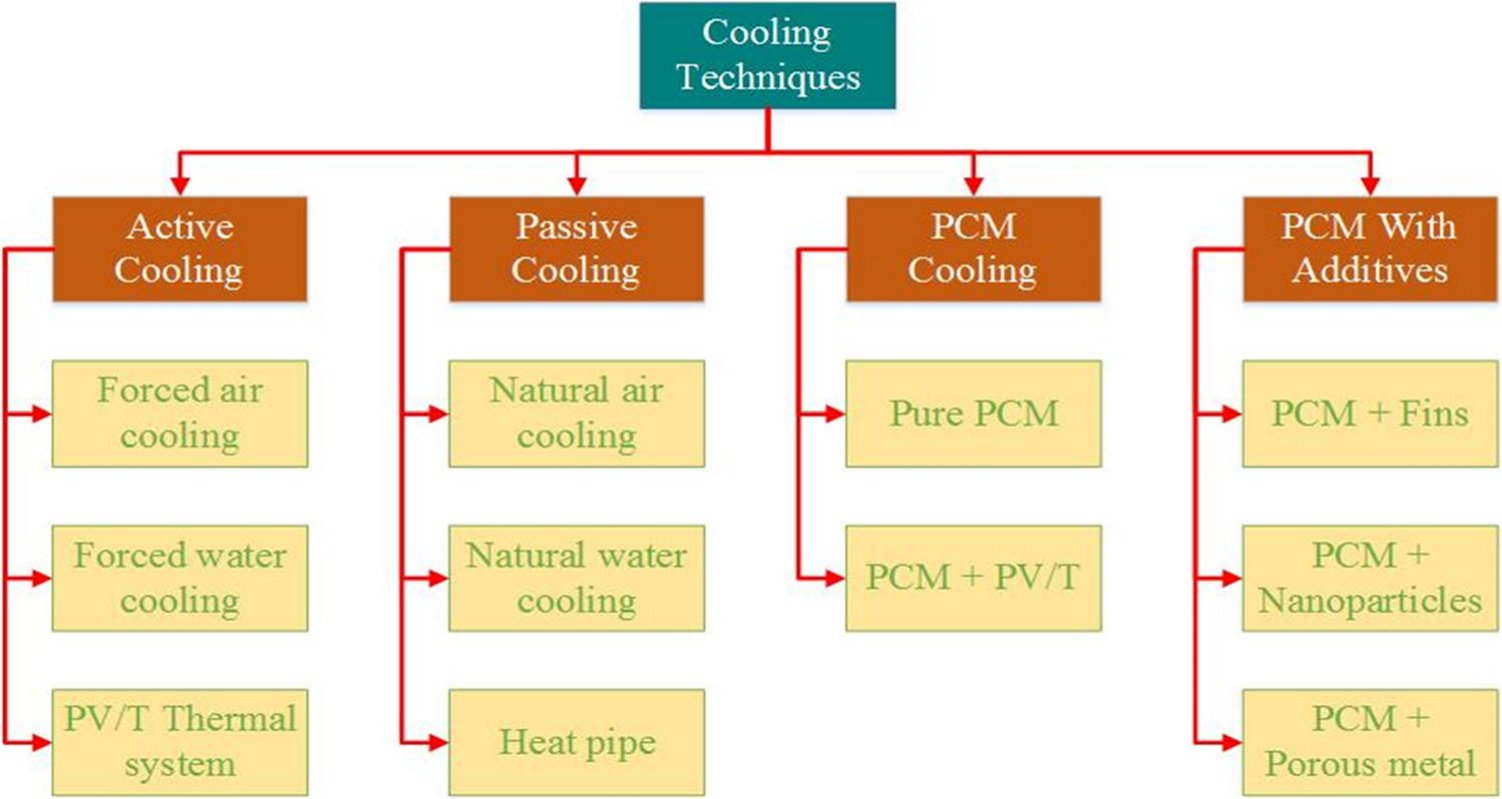
The different cooling techniques

The performance of the PV panels worsens due to increasing its operating temperature. Therefore, to maintain the electrical performance of the PV module at an acceptable level, it is essential to utilize an appropriate cooling technique to lower its surface temperature, thereby prolonging its lifetime (Shastry and Arunachala, 2020). Most researches are carried out to minimize the effect of high temperature on the PV module’s electrical efficiency in order to make the power produced from the PV module able to challenge the non-renewable sources of energy. Different types of cooling methods used in PV cooling are reviewed in the following sub-sections and are summarized in Fig. 3

### Active cooling

This method relies on another mechanical device to pump the water or air necessary for cell cooling. For this, it needs a continuous source of power which relatively has a large ability to cool the solar cells (Abo-Elfadl et al., 2020b; Hassan et al., 2021). The power used in the fan or pump is deducted from the power produced from the cell, reducing the net output power produced from the PV cells (Abdulmunem et al., 2020). This method is divided into two basic types: first, forced air cooling, and second, forced water cooling, and many studies have been conducted on the two types, which will be reviewed in the next sections.

#### Forced air cooling

**Fig. 4:**
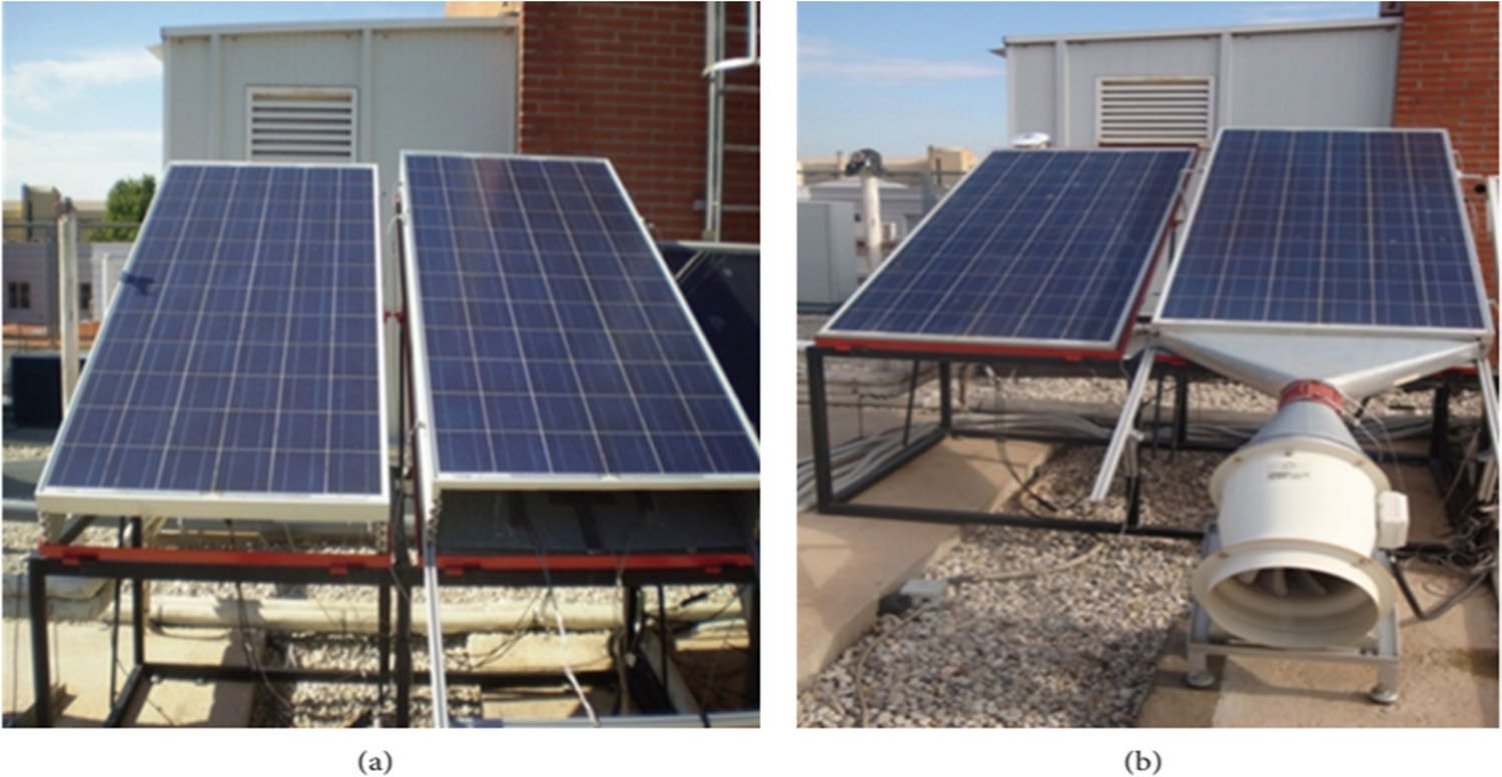
Experimental setup. Left panel is a reference, and the right panel is above a steel plate, creating an air channel underneath it. **a** First setup (natural convection). **b** Second setup (forced convection) (Káiser and Zamora, 2013)

Amelia et al. (2016) performed a practical experiment to use a number of DC fans directed at the back of the cell to cool it. It was noticed that with the increase in the number of fans, the cooling rate increased, and the power produced by the cell increased, but the power required to operate these fans also increased. The use of one fan increased the power produced by 12.93%; when the number of fans is raised to 2, 3, and 4, the power increased to 37.17%, 41.28%, and 44.34%, respectively. Káiser and Zamora (2013) conducted an experiment to compare the natural convection and forced convection in photovoltaic cooling. In their setup, he used two photovoltaics; one is a reference, and the other has a steel plater under it to create an air channel underneath it. The first study let the natural air circulate through the channel to cool the photovoltaic by natural convection. The second study uses a centrifugal fan to force the air inside the channel to cool photovoltaic by forced convection, as shown in Fig. 4. The results investigated that the forced convection produced a drop in surface temperature = 15 °C and an increase in the electrical power = 15% comparing to the natural convection.

**Fig. 5:**
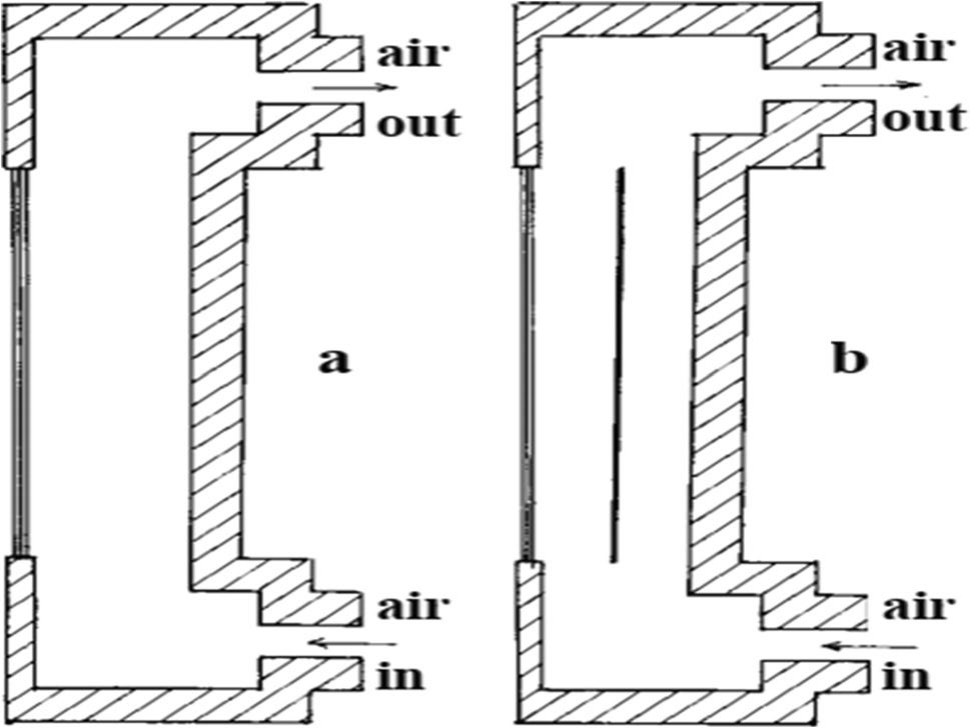
Cross-section of experimental models (Tripanagnostopoulos, 2001)

Tripanagnostopoulos (2001) experimented to enhance the photovoltaic performance by cooling it by forced air convection. In their study, they used three photovoltaic cells: the first is reference, the second is modified by design an air channel at its back surface, and the third is the same as the second but modified by adding a thin sheet metal at the center of the channel as shown in Fig. 5. The results indicated that the higher the flow rate, the higher the increase in electrical efficiency and reduction in surface temperature, and the maximum reduction in temperature occurred was 7.8% and 9.5% for the module with air channel only and the module with an air channel and thin metal respectively. A study to improve the performance of PV module using forced air cooling technique has been presented by Sajjad et al. (2019). The results presented in this study was compared with PV modules without cooling. Based on the comparison, it was shown that using air cooling technique resulted in 7.2% and 6% higher electric efficiency and power ratio, respectively.

**Fig. 6:**
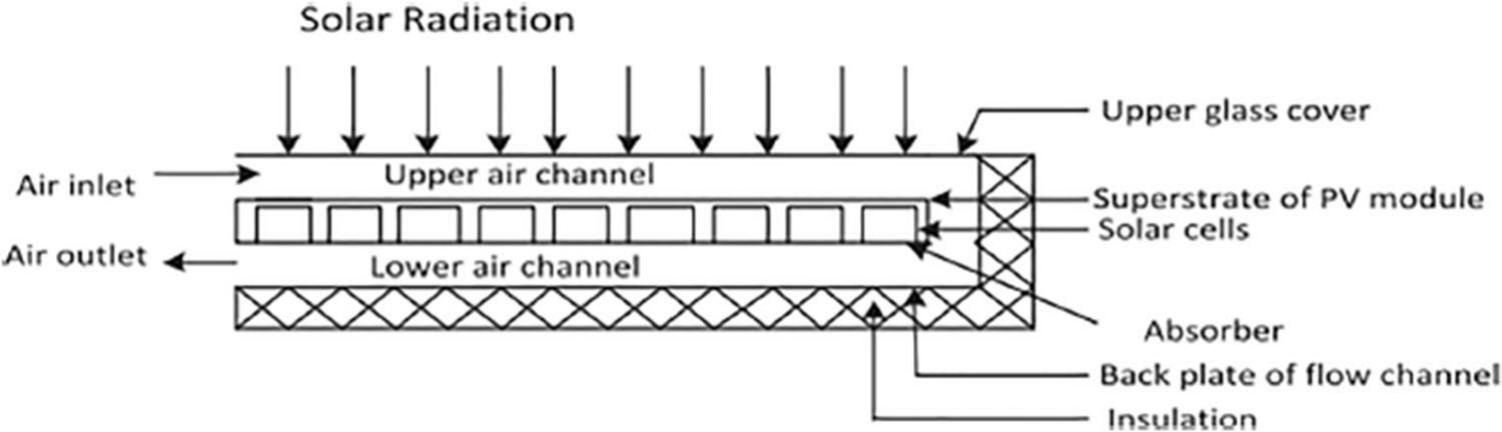
Air-cooled PV panel (Kumar and Rosen, 2011)

**Fig. 7:**
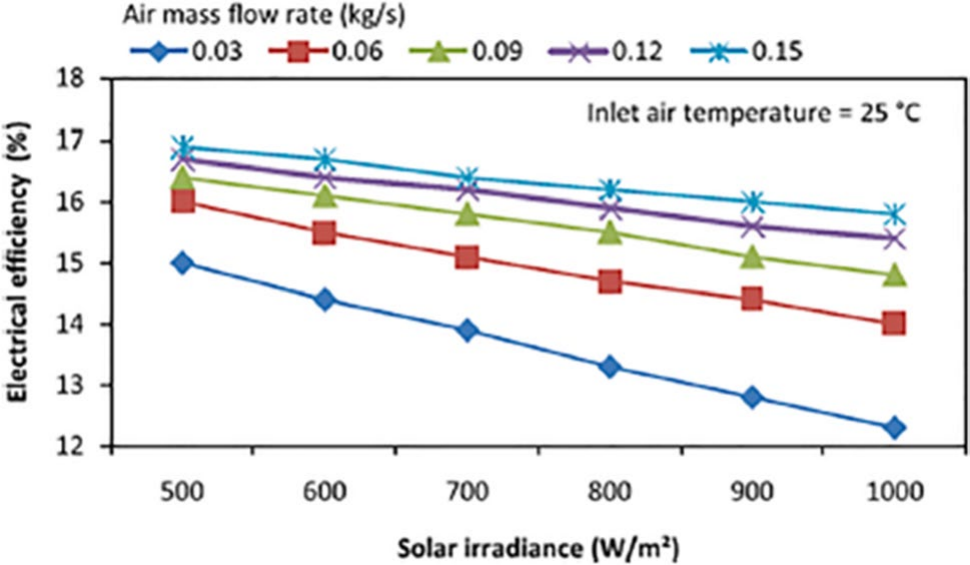
Effect of mass flow rate on the electrical efficiency of air-cooled PV (Kumar and Rosen, 2011)

Due to low convective heat transfer capacity of air, fins are employed profusely in this thermal management approach (Abo-Elfadl et al., 2021a, b). For instance, Kumar and Rosen (2011) designed a system, shown in Fig. 6, for cooling a PV panel by employing the air flow. In their work, different parameters affecting the efficiency of the system, such as mass flow rate of air and solar irradiation, were investigated. In addition, the effect of inserting fin on the thermal and electrical efficiencies of the system was analyzed. According to their observations, adding fins resulted in 10.5% and 15% enhancement in the electrical and thermal efficiencies of the system, respectively. Moreover, increment in air mass flow rate from 0.03 to 0.15 kg/s led to significant increase in the electrical efficiency as illustrated in Fig. 7. As presented in Fig. 7, increase in solar irradiation reduces the efficiency of the cell, which is mainly due to its temperature increment.

Sahay et al. (2015) reported a newly developed method named as ground-coupled central panel cooling system (GC-CPCS). The projected scheme serves to cool the solar panels by forced convection of air driven by a blower, the blower being run by another dedicated PV panel. Air flows through a ground-coupled heat exchanger and decreases its temperature. The cooled air soothes the solar panels while passing beneath them. The researchers installed nine solar panels of 100 W each. The air is flown by a single blower and the cold air is distributed to each solar panel through the pipe. Nozzles are attached to the pipes in order to ensure that streamline flows in desired directions. The author reported marked improvement in conversion efficiency using GC-CPCS.

**Fig. 8:**
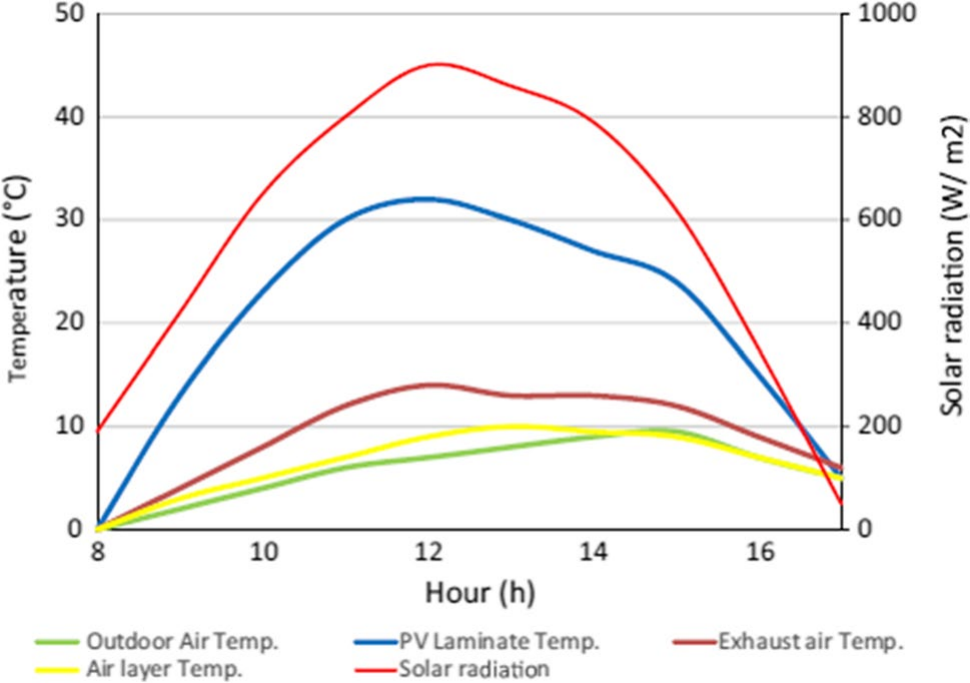
Temperature of PV module, air layer and exhaust air (Kim et al., 2014)

Experimental performance study of a PV thermal air collector has been conducted by Kim et al. (2014). Forced ventilation of air at a rate of 240 m^3^/h was maintained by means of a fan installed in the exhaust air pipe. It is evident from Fig. 8 that temperature of the PV module could be retained low at 12–32 °C because of the heat carried away by the exhaust air which reached 3.5–14 °C while the ambient temperature was 1.6–9.5 °C. So, the heated air from the air collector had around 5 °C elevated temperature than the ambient air. The authors reported that the thermal and electrical efficiency of the system were about 22 and 15%, respectively. The electrical efficiency of the PV panel at STC is 15.46%, which indicates that the performance of the hybrid photovoltaic thermal (PVT) air collector is very near to the PV the only system.

#### Forced water cooling

**Fig. 9:**
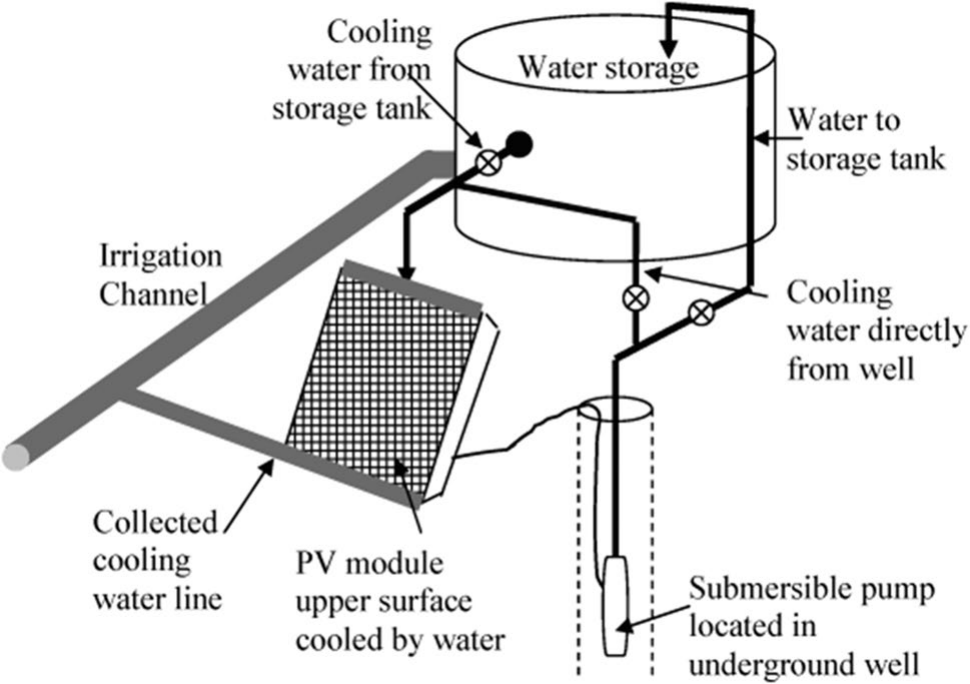
The experimental setup of the experiment (Odeh and Behnia, 2009)

Krauter (2004) used a pump to force the water through holes installed at the top of the PV cell’s surface. He was able to cool the surface of the PV cell and overcome the dust and drift on the surface of the cell, which reduces its efficiency. He finally succeeded in increasing the electrical efficiency of the cell by 9%. Odeh and Behnia (2009) experimentally cooled the PV solar cell by pumping water on the cell’s surface, as Fig. 9 shows, and record the results over the year’s seasons. The results showed an increase in the cell output up to 15% in the hotter summer season, and the average increase in the cell output as a result of the cooling process throughout the year reaches 5%. The presence of water on the cell’s surface has led to cooling the cell’s surface, removing the accumulated dust on the cell’s surface, and increasing the solar radiation due to the refraction of the solar ray in the water layer.

**Fig. 10:**
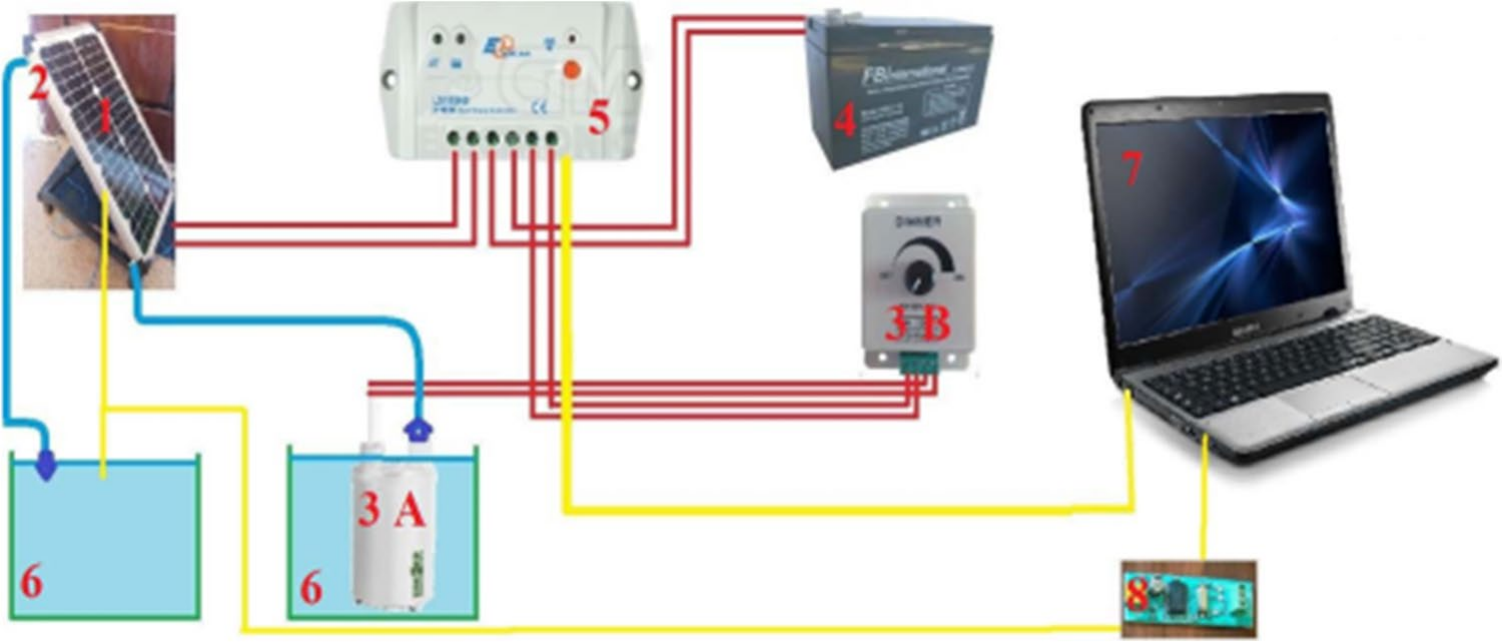
Diagram of the experimental setup: 1, PV panel; 2, aluminum radiator; 3A, submersible pump; 3B, dimmer; 4, rechargeable battery; 5, battery charger; 6, water tank; 7, laptop (Colţ, 2016)

Colţ (2016) conducted an experiment to cool the photovoltaic by circulating water at its rear surface. In his setup, he used an aluminum radiator as a heat exchanger to extract the heat from the photovoltaic, as shown in Fig. 10. The results indicated that the photovoltaic surface temperature decreased by 32%, and the electrical efficiency increased by 57%. A comparison study of cooling techniques for PV module with reflectors has been carried out by Kabeel et al. (2019) under Egyptian climate conditions. Three different cooling techniques, namely forced air, water cooling, and combination of forced air/water cooling were considered in the study. The experimental results showed that the water cooling was the best cooling option for PV module under Egyptian climate conditions. An experimental work has been carried out to investigate the effect of geothermal air cooling on the PV module behavior by Elminshawy et al. (2019) where the results showed that both power and efficiency of PV module were improved.

**Fig. 11:**
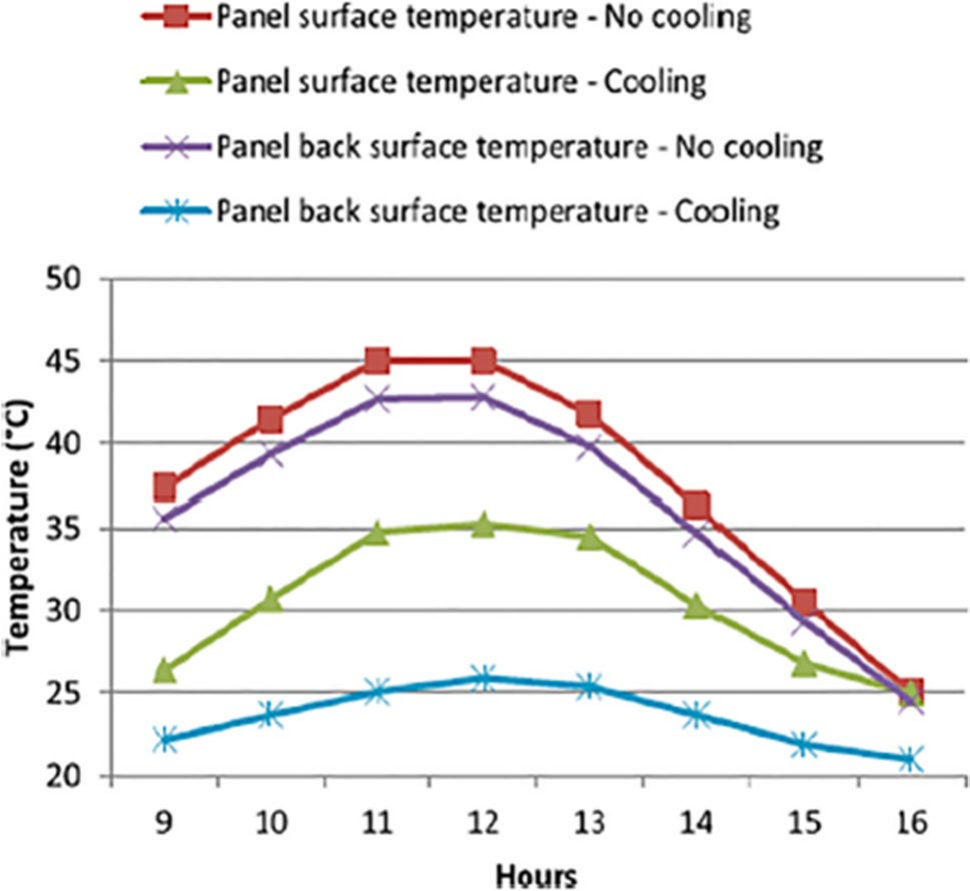
Effect of water cooling on the cell temperature (Bahaidarah et al., 2013)

**Fig. 12:**
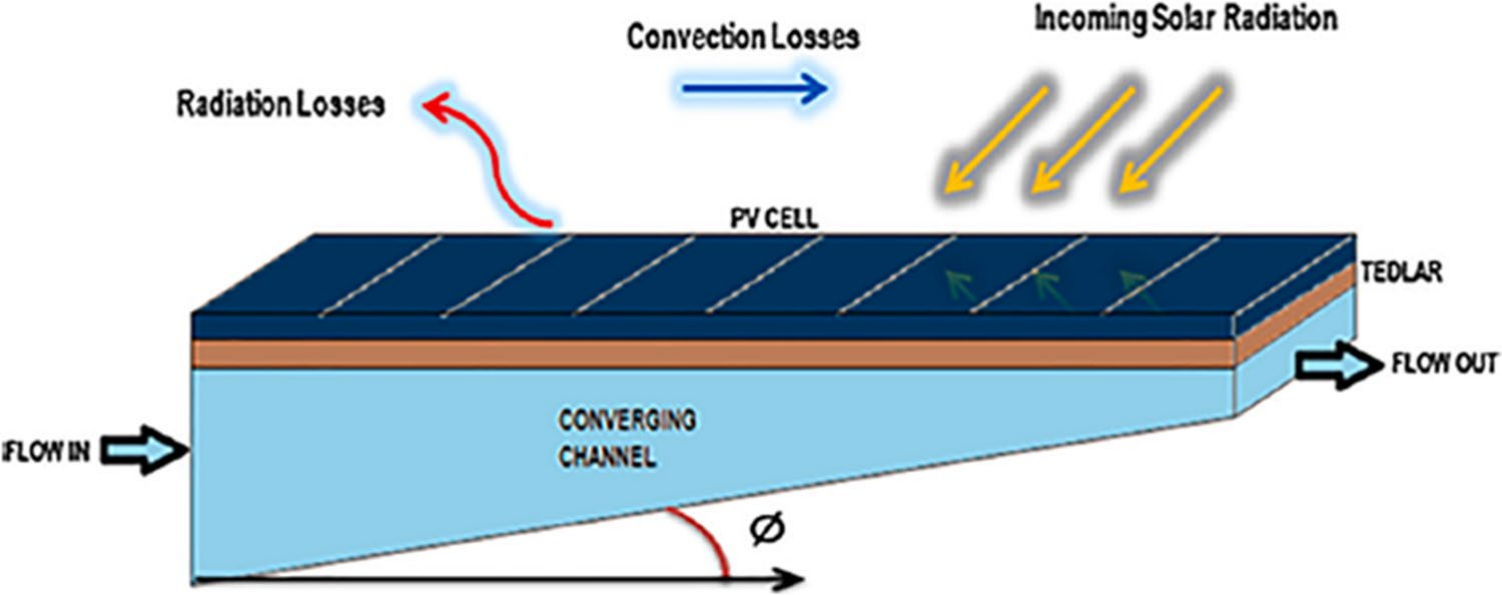
Converging channel used for PV cooling (Baloch et al., 2015)

Bahaidarah et al. (2013) employed water cooling for PV cells as shown in Fig. 11. The comparison of the temperature in the tested modes, including the case with no cooling and the case with water cooling, revealed the efficiency of this approach in cooling down the PV as presented in Fig. 11. According to the results, using active water cooling for PV modules can lead to approximately 20% reduction in module temperature which translates to about 9% efficiency enhancement. In addition to the improvement in the electrical generation efficiency of PV, by applying this method, the extracted heat can be used for different purposes, which means increase in collected energy. Several novel ideas are tested out in liquid cooling of PVs with the goal to achieve more uniform temperature in the cells such as converging channel tested by Baloch et al. (2015). The designed shape of channel is shown in Fig. 12. Due to mass conservation in converging channels, reduction in the cross-section leads to velocity increment, which enhances forced convection; consequently, the temperature profile is more uniform. In order to find the most appropriate converging angle, seven angles were modeled and assessed. The considered angles varied from 0° to 10°, while the mass flow rate and outlet cross-section were kept constant. The most favorable results, in terms of temperature uniformity, were obtained at 2° converging angle. According to the thermal analysis of the system, by using converging channels, the PV temperature can be reduced from 71.2 to 45.1 °C and from 48.3 to 36.4 °C in a typical hot day in June and a cold day in December, respectively. In addition to thermal evaluation, the cooled and uncooled systems were assessed economically. According to the results, levelized cost of electricity (LCOE) was reduced form 1.95 $\frac{ \in }{KWh}$ to 1.57 $\frac{ \in }{KWh}$ by employing the cooling approach.

#### PV/T thermal system

It is also possible to use the active cooling approach in cooling the cells and transferring the heat produced from the cell to the cooling medium such as water. This hot water is used for other additional uses. This type is symbolized by PV/T systems (Ozgoren et al., 2013), experimentally compared between two cells, the first without any additions and the other by adding a pipe at the back of the cell through which the water passes, which cools the cell. At the same time, the water is also used as hot water for various uses. The results indicated that the PV cell conversion efficiency rose from 11.5 to 13.6%, and the thermal efficiency value reached 51%. Teo et al. (2012) also conducted a practical experiment to cool the cell using the (PV/T) thermal system by pumping air to the air duct at the back surface of the cell and controls the amount of air with a sensor linked to the temperature of the PV cell surface. The comparison results proved that the conventional PV surface temperature reaches 68 °C and its conversion efficiency was 8.6%. In contrast, in the case of a cooled cell, the cell surface temperature decreases to 38 °C and the conversion efficiency reach up to 12.5%. The results also proved that the optimum airflow rate is 0.055 kg/h. The higher airflow rates do not constitute an additional improvement in the cooling process but rather increase the power required to operate this fan.

**Fig. 13:**
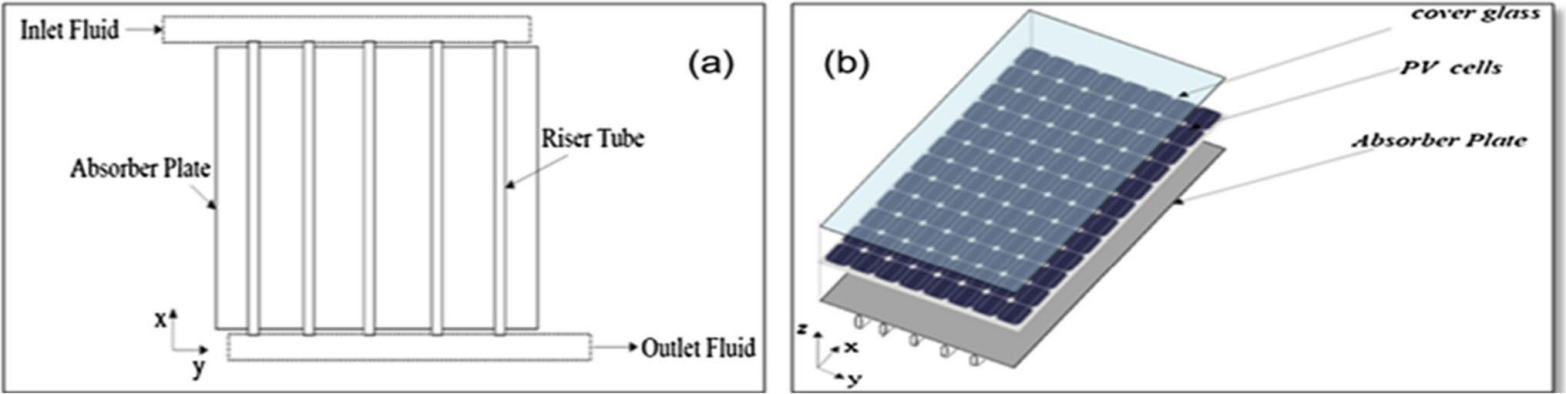
(a) Sketch of the model, (b) schematic of the setup. (Khanjari et al., 2016)

Khanjari et al. (2016) numerically studied the cooling of photovoltaic panels by PV/T system. They compared the uses of three types of fluids: pure water, Alumina water nanofluid, and Ag water nanofluid. Their model includes an absorber plate and riser tube to consider the convection and conduction heat transfer mechanisms, as shown in Fig. 13. The results indicated that the electrical efficiency increased by 1.83% and 3.9% for Alumina water and Ag water, respectively, compared to the pure water, while the overall efficiency increased by 4.26% and 11.54% for Alumina and Ag water, respectively, compared to the pure water.

**Fig. 14:**
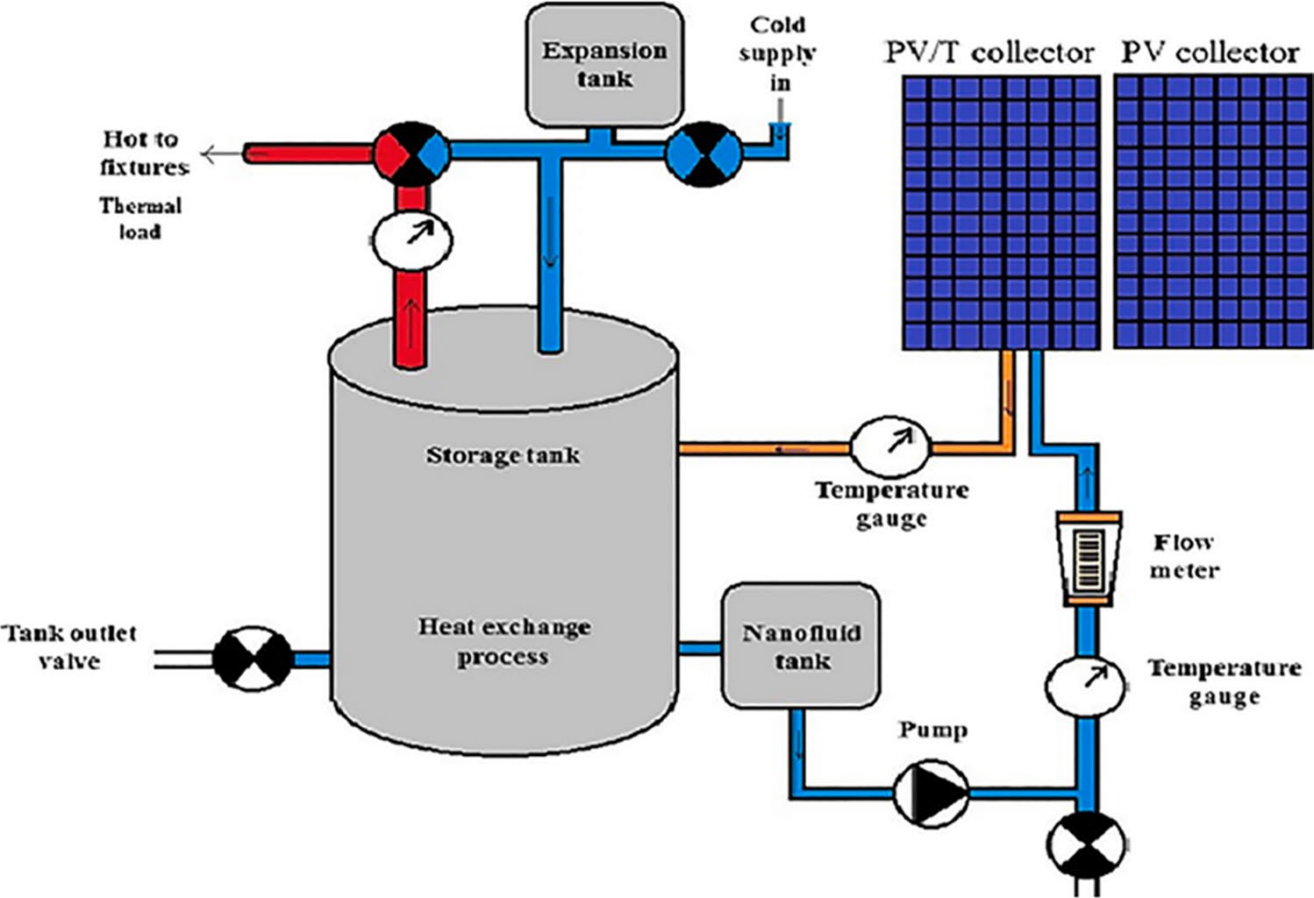
Schematic of nanofluidic PV/T system (Al-Waeli et al., 2017)

**Fig. 15:**
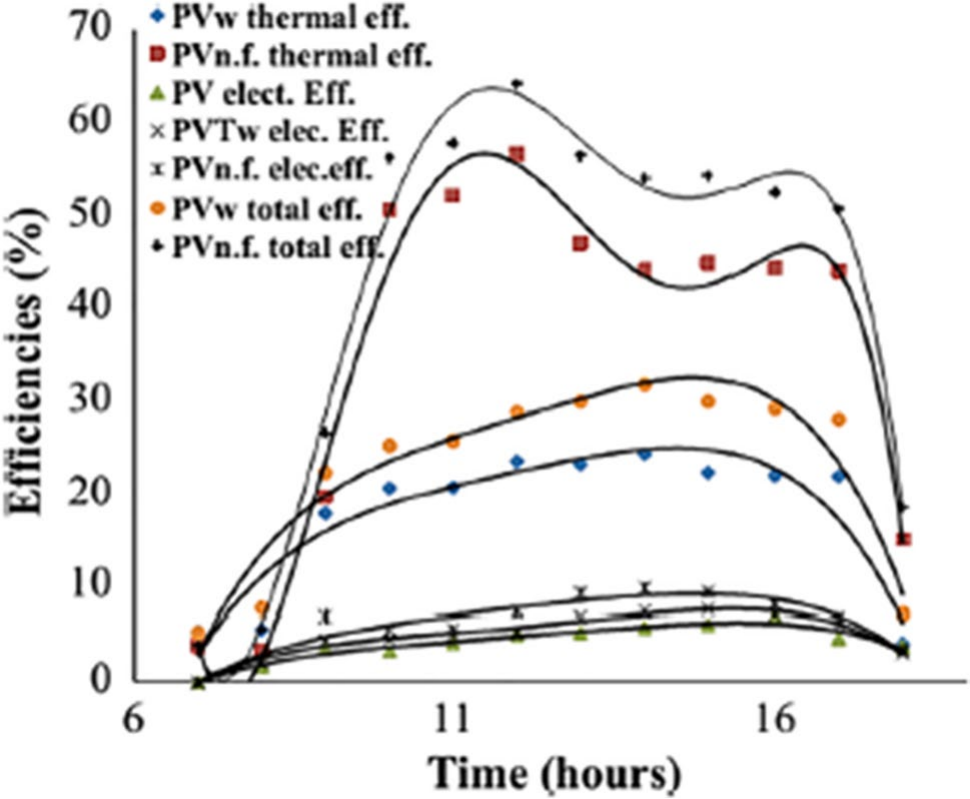
Efficiencies of the system at different conditions (Al-Waeli et al., 2017)

In a study carried out by Al-Waeli et al. (2017), SiC/water nanofluid was employed in a PV/T system as shown in Fig. 14. In the first step of their study, thermal features of nanofluids in different concentrations including 1%, 1.5%, 2%, 3%, and 4% were measured and compared. The increment thermal conductivities of the nanofluid in the mentioned concentrations were 2.5, 2.9, 4.5, 8.3, and 8.4%, respectively. According to these values, 3wt% concentration was selected for testing in the system since limited improvement in the thermal conductivity was observed in higher concentrations. The outcomes of this research demonstrated that employing the nanofluid leads to up to 24.1% increase in electrical efficiency of the system in comparison with using PV without cooling system. The generated power in the case of using nanofluid was 57% and 25.6% greater compared with the PV-alone and PV water-cooled system, respectively. In addition to electrical efficiency, the thermal efficiency of nanofluid-cooled systems was higher than the water-cooled one by approximately 100.19%. The obtained efficiencies of the system are represented in Fig. 15.

Wu et al. (2018) carried out a numerical study on PV/T system having a water channel fitted above the PV panel. They developed a 3D physical and mathematical model to access the effect of different parameters like height of the channel, cooling water inlet temperature, the mass flow rate of water, and intensity of radiation on the performance of PV/T system. The thermal and electrical, energy, and exergy efficiency of the system was also calculated. For validation of the mathematical model, results of the numerical analysis were compared with experimental data of Erdil et al. (2004) who have built and tested the performance of a system of similar configuration. Results showed that the system’s thermal efficiency is greatly affected by the mass flow rate of water and both thermal and electrical efficiency showed an increasing trend with the mass flow rate. The total exergy efficiency had a maximum value of 13.8% at an optimal flow rate of 0.003 kg/s. Any rise in mass flow rate above the optimal value resulted in a reduction in the total exergy efficiency. They found the optimum height of the channel to be 5 mm, from the total exergy efficiency viewpoint. They found that with increasing radiation intensity, exergy efficiency increases. The results also indicated that the panel’s electrical efficiency decreases by installing a water channel above the PV panel, but results in improved thermal performance, as a result when compared to a conventional system which has a higher overall efficiency.

Zanlorenzi et al. (2018) proposed a novel active cooling technique using water as a coolant for performance enhancement of the PV module. They designed and developed a hybrid PV/T collector that simultaneously converted solar energy into electrical and thermal energies. The initial design and prototype development of the hybrid module was done in Solid Works simulation software. A serpentine tube was fixed to the rear surface of a PV module having 250-W power output, and an electric pump which consumed 2.66-W power was used to maintain the circulation of water inside the serpentine. The experimental setup was installed in Panama, Brazil, and to know the influence of solar radiation intensity on PV module, data were recorded in three intervals of time (a) 8:00 am to 10:59 am, (b) 11:00 am to 2:59 pm, and (c) 3:00 pm to 5:59 pm. The experimental results showed a decrease of 8.83 °C in the maximum effective temperature of the hybrid module in relation to the conventional module. The mean electrical efficiency of the hybrid and the original module was observed to be 15.20% and 13.95%, respectively, which shows a gain of 1.25% in the electrical efficiency. The hybrid module was found to produce 8.22% more energy than the conventional module. The proposed design improved the thermal performance of the hybrid system with 23.5% maximum thermal efficiency. They concluded that the proposed hybrid module not only increases the electrical power output but also improves the life of the PV module, by decreasing the maximum effective temperature which leads to overheating of the module and reduces their life.

#### Discussion

In the field of active cooling, optimization to obtain uniform temperature distribution has been achieved by improved heat exchanger designs. Multipass serpentine field heat exchanger was shown to be operating with less temperature variation. Also the use of variable flow rate of coolant can increase temperature uniformity across the surface of the PV panel.Adding nanoparticles to liquid is a promising option which can attain a large amount of heat removal rates. They were reported to cool the temperature of PV panels in the range of 20–45 °C for concentrated systems.Cooling with thermal system PV/T was also found to be effective in increasing the efficiency and lowering the temperature. Furthermore, temperature variation across PV surface was reduced to 3–10 °C by applying PV/T. The most important disadvantage of active cooling is the power required to operate the compressor or pump used in the cooling process.

### Passive cooling

This method is characterized by the absence of the need for a variable cost that only requires a fixed cost to establish the cooling system and does not require power to operate this system. This method is represented by natural cooling with water or with air and heat pipe, but it improves the efficiency of the PV cell by a small percentage.

#### Natural air cooling

**Fig. 16:**
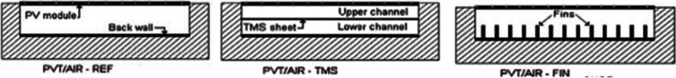
The three cross-sections of (Tripanagnostopoulos and Themelis, 2010) modules

Tripanagnostopoulos and Themelis (2010) did three modules for cooling PV solar cells through natural air. The first module contains an air duct at the back of the cell; the second contains an air duct like the first module, by inserting a thin metal sheet (TMS) in the middle of this path to increase the cooling process; and the third contains metal fins in the back of the cell to increase the surface exposed to air and thus increase the rate of cell cooling as shown in Fig. 16. The results indicated the highest efficiency among the three modules is the third module (fins), followed by the second module (TMS), and then the first.

**Fig. 17:**
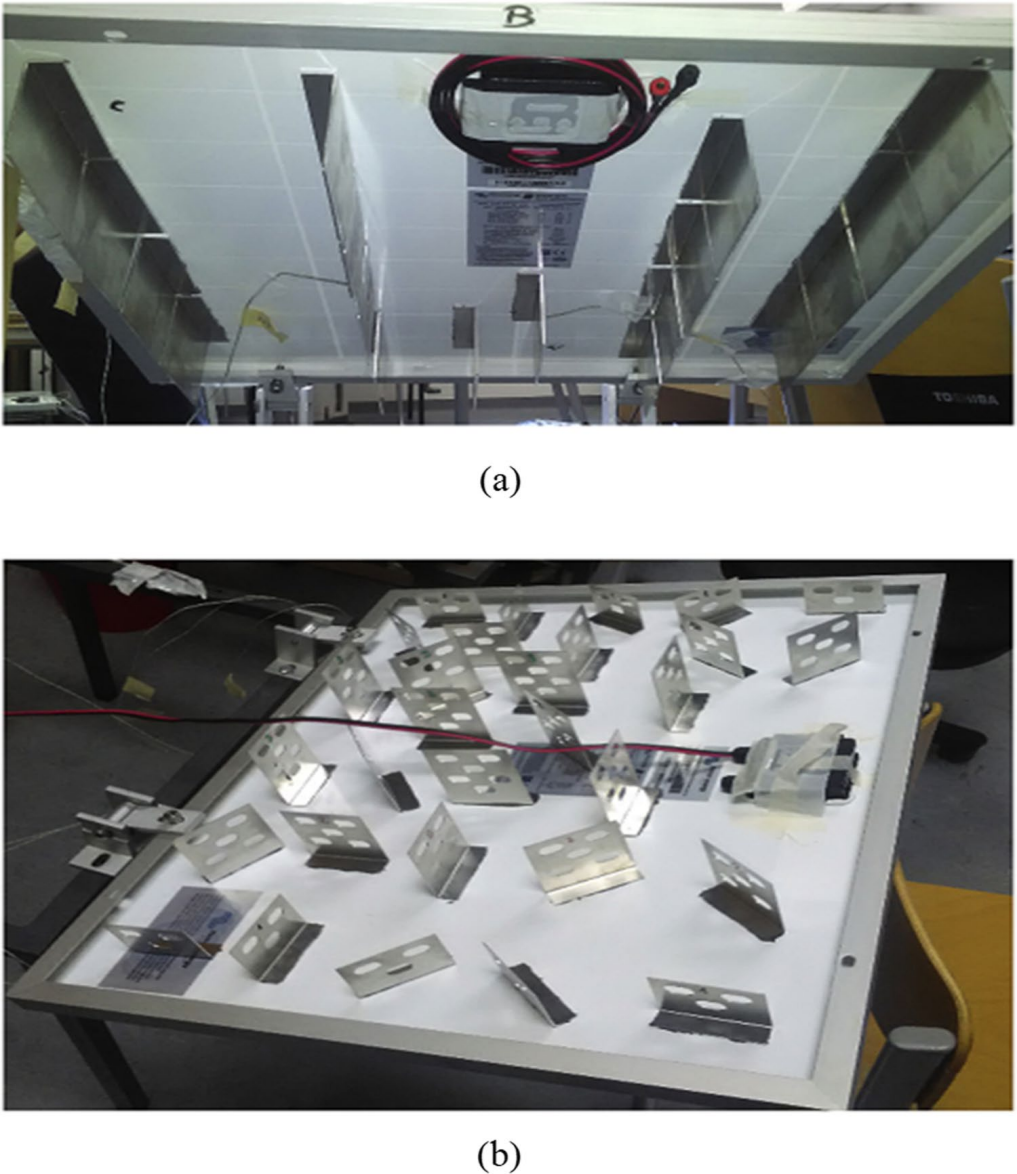
**a**, **b** A photograph of modules belongs to Grubišić-Čabo et al. (2018)

Bayrak et al. (2019) conducted a practical experiment to cool the cells using fins and used ten different geometries for the fins. The maximum temperature difference they obtained was 3.39 °C under the radiation of 772 W/${\mathrm{m}}^{2}$. This difference led to an increase in the power produced by 6.84 W. In this case, the value of the electrical efficiency was 11.55%, and the exergy efficiency was 10.91%. Grubišić-Čabo et al. (2018) did a practical experiment to cool the PV solar cell using fins and created two modules. The first contains aluminum fins affixed longitudinally and regularly to the back surface of the cell, as shown in Fig. 17a, while the second module contains aluminum fins attached to the back surface of the cell randomly and irregularly, as shown in Fig. 17b. It was discovered from the results that the second module gives better results than the first, as the second module was able to raise the efficiency of the cell by 2%.

El Mays et al. (2017) experimented PV panel with heat sink of the parallel-finned aluminum plate in the frontside. The presence of a finned plate led to a decrease in front temperature by an average of 6.1 °C, and hence, conversion efficiency and output power were emphasized by 1.75% and 1.8 W, respectively, compared to the un-finned panel. Also, utilizing aluminum heat sink enhanced the performance of PV under natural convection, as resulted from the study conducted by Cuce et al. (2011) under different solar radiation intensities. The increase in energy, power conversion, and exergy efficiency utilizing finned heat sink reached 9, 13, and 20%, respectively, at 800 W/m^2^.

**Fig. 18:**
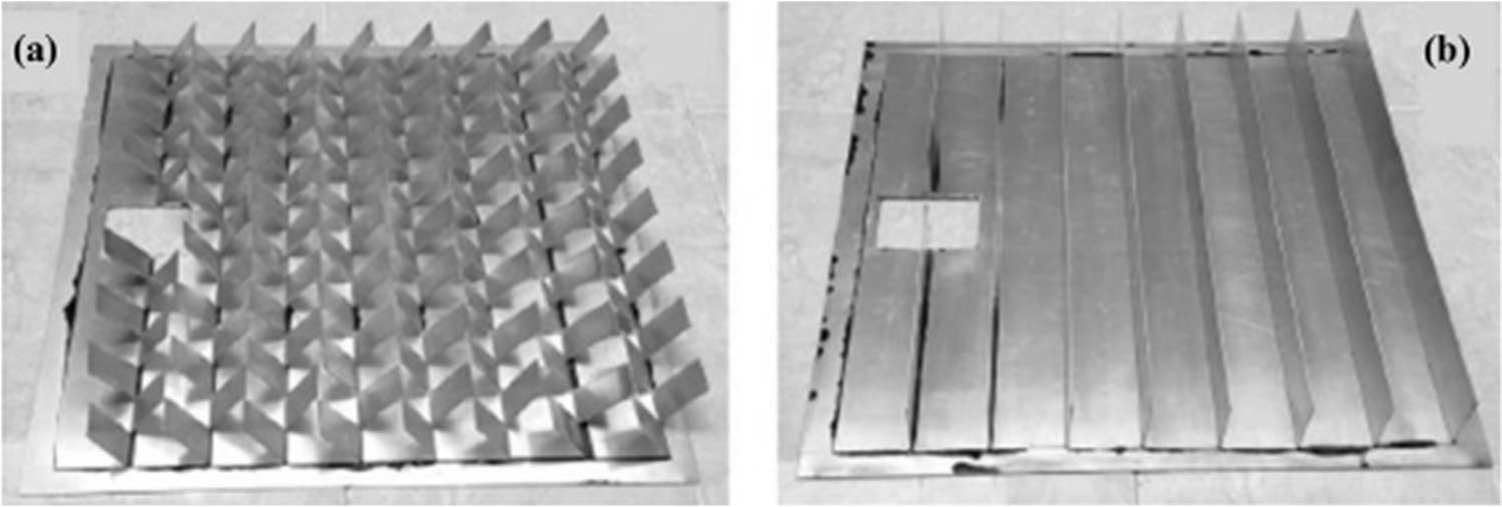
Heat sinks: **a** segmented and **b** conventional (Hernandez-Perez et al., 2020)

Moreover, using the different orientation of fins was considered by Hernandez-Perez et al. (2020) through investigating aluminum heat sinks: ordinary straight fins and other with inclined segments, as shown in Fig. 18. The heat sinks were first evaluated at normal operational conditions using CFD software to state heat sink of the best performance for further experimental investigation. It was found that segmented heat sink showed better performance with lower pressure losses as it reduced velocity losses increasing turbulence, and hence convective heat transfer was enhanced. Both numerical simulation and experimental results showed good agreement as maximum temperature reduction was about 9.4 and 10 °C, respectively, with conversion efficiency enhancement of ~ 4% at peak hours of solar intensity (800:1000 W/m^2^). Based on the results, an increase in capacity per square meter of 6 W can be obtained.

**Fig. 19:**
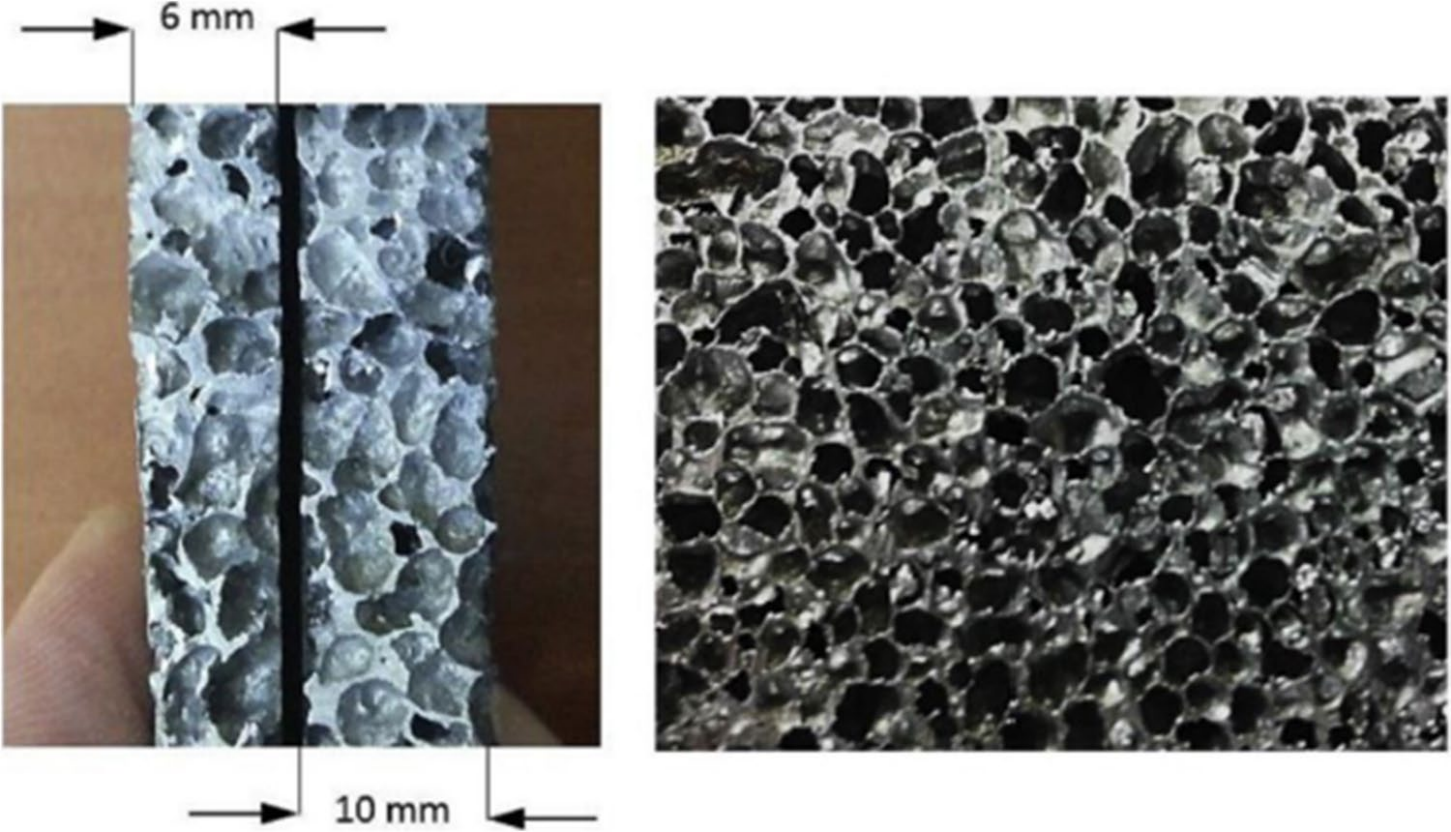
Closed-cell aluminum foams (6 mm, 10 mm, and general view) (Selimefendigil et al., 2018)

In addition, Selimefendigil et al. (2018) performed experimental and forecasting studies on the efficiency of PV panels with closed-cell porous fins made of aluminum metal foam with very high porosity characteristics of 75:95% as shown in Fig. 19. Furthermore, a multi-input dynamic system was proposed, based on artificial neural networks for finned and un-finned PV based on experimentally measured data. As resulted, porous fins, specially 10 mm, enhanced the performance with maximum power difference of 7.26 W. Both experimental and estimated data showed good agreement and the developed dynamic neural networks model proved its ability of performance predictions of these systems.

#### Natural water cooling

**Fig. 20:**
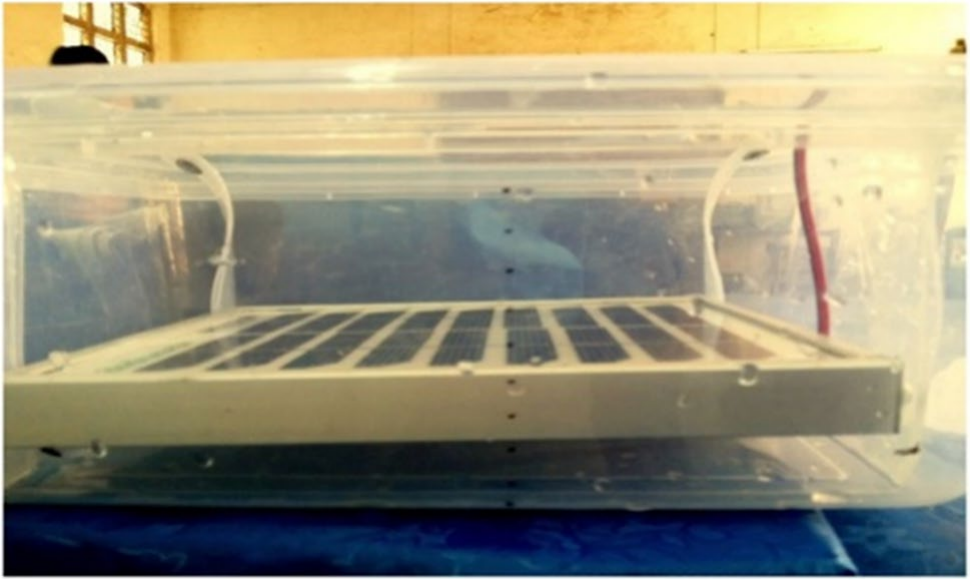
Panel immersed in water (Mehrotra et al., 2014)

Wilson (2009) created a practical experiment in which he used the available water at an appropriate head so that it would pass on the back of the PV cell without the need for a pump. The power required for the flow of water from top to bottom is the energy available in the water due to the difference in the hydraulic head. The surface temperature of the PV cell was reduced from 60 to 30 °C, and an increase in the conversion efficiency of the cell up to 12%. Another method is used to cool the cells by dipping the PV solar cell in a water basin. Mehrotra et al. (2014) did an experiment to cool the PV solar cell by dipping it in different depths of water, as shown in Fig. 20. The results proved that the greater the water depth, the lower the surface temperature of the PV solar cell, and thus PV cell efficiency increases. The highest electrical efficiency obtained was 4.76% at a depth = 1 cm, with an increase in the electrical efficiency = 17.8% compared to the PV solar cell at the water’s surface.

**Fig. 21:**
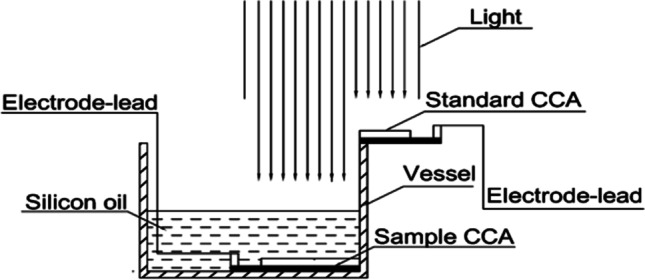
The schematic diagram of Xin et al. (2015)

Xin et al. (2015) conducted an experiment to cool the concentrating photovoltaic (CPV) systems by immersing them in dimethyl silicon oil with thickness = 1 mm: 30 mm. Figure 21 shows the schematic diagram of its setup; they studied the effect of the immersing fluid and its thickness on the electrical performance of the CPV systems. The results proved that the electrical efficiency and output power of the CPV at silicon oil = 1 mm in thickness, increased by 2.6% and 2.7%, respectively. Also, the higher the silicon oil thickness, the lower the electrical performance, and above silicon oil thickness of 6.3 mm, the electrical efficiency and the output power of the CPV without immersing higher than the electrical efficiency and the output power of the CPV with immersing.

Saxena et al. (2018) studied the water-cooling impact under continuous and intermittent flow regimes, using a laboratory test-rig., under solar radiation range from 87.38 to 359.17 W/m^2^. For intermittent one, various mass flow rates (3, 5.3, and 6.2 L/min) were utilized through which, all, energy production was enhanced by 18% w.r.t uncooled system. On the other hand, for continuous feeding, the flow rate was maintained at 0.6 L/min resulting in 29% power enhancement. Hence, the proposed technique of PV module cooling was recommended to be built-in with the water supply arrangement for house applications.

Han et al. (2011) carried out an experimental investigation to predict the performance of Si concentrating photovoltaic solar cells dipped in four different kinds of liquids. The experiments were conducted on a solar cell of 40 mm width, 50 mm length, and an aperture area of 19.5 cm^2^, made up of mono-crystalline silicon cells with silicon dioxide anti-reflection coating. De-ionized (DI) water, dimethyl silicon oil, isopropyl alcohol (IPA), and ethyl acetate were chosen as immersion liquids. To understand the effect of thickness of the liquid film directly above the cell surface, two separate tests were conducted one with 1.5 mm and other with a liquid layer thickness of 9 mm. The 9-mm test was also used to understand the influence of absorption of the incident light by different liquids. The tests were conducted at 30 Sun and 25 °C. Results showed in comparison to *I*_sc_ and *V*_oc_ of CPV solar cells in the air, the *I*_sc_ and *V*_oc_ of the cells immersed in liquids of 1.5-mm thickness were larger, although the overall variation in *V*_oc_ was comparatively less than that of the *I*_sc_. Maximum change of 15.5% in *I*_sc_ was recorded for the cell immersed in IPA. The largest change in efficiency was observed to be 15.2% for the cell immersed in IPA with 8.5% being the minimum change for the cell in DI water. The test results clearly demonstrated that with an increase in liquid layer thickness, the degree of improvement in cell efficiency decreases due to increased absorption of the incident light.

#### Heat pipe

**Fig. 22:**
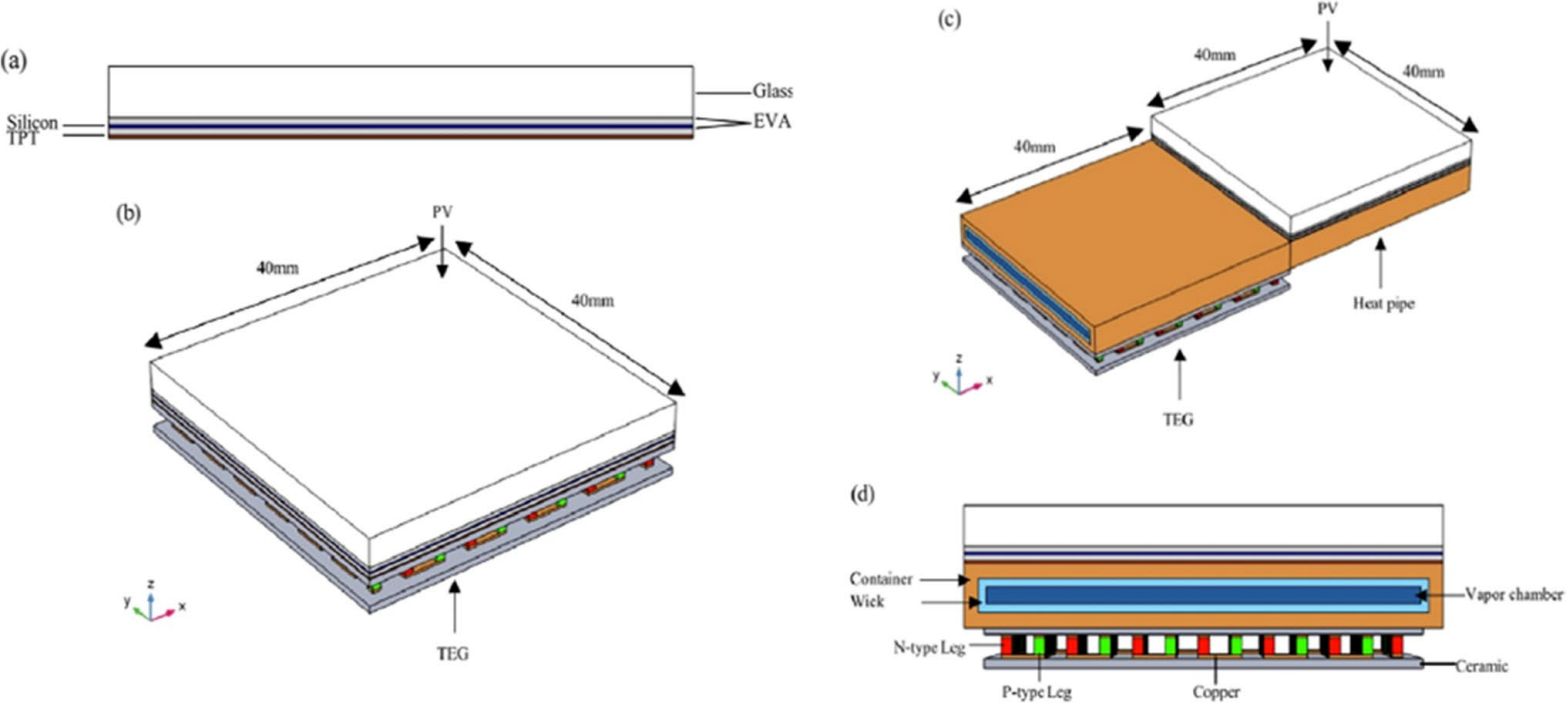
The schematic diagram of Shittu et al. (2019). **a** PV, **b** PV with TEG, **c** PV with TEG and heat pipe, **d** PV with TEG and heat pipe side view

The flat heat pipe is a vapor–liquid phase change equipment that works as a small refrigeration cycle. It has two parts, one is maintained cool (evaporative) and the other hot (condenser), so it can be used in cooling electrical devices such as photovoltaics. It has advantages such as no moving parts, compact structure, and high heat transfer, while its disadvantage is required high cost. Shittu et al. (2019) conducted an experiment to improve photovoltaic performance by using a heat pipe. They created three modules, the first is a PV used as a reference, the second is a PV coupled with thermoelectric TEG, and the third is a PV integrated with TEG and heat pipe, as shown in Fig. 22. The results indicated that the electrical efficiency of the third module is 1.47% and 61.01% higher than the electrical efficiency of the second and first modules, respectively.

**Fig. 23:**
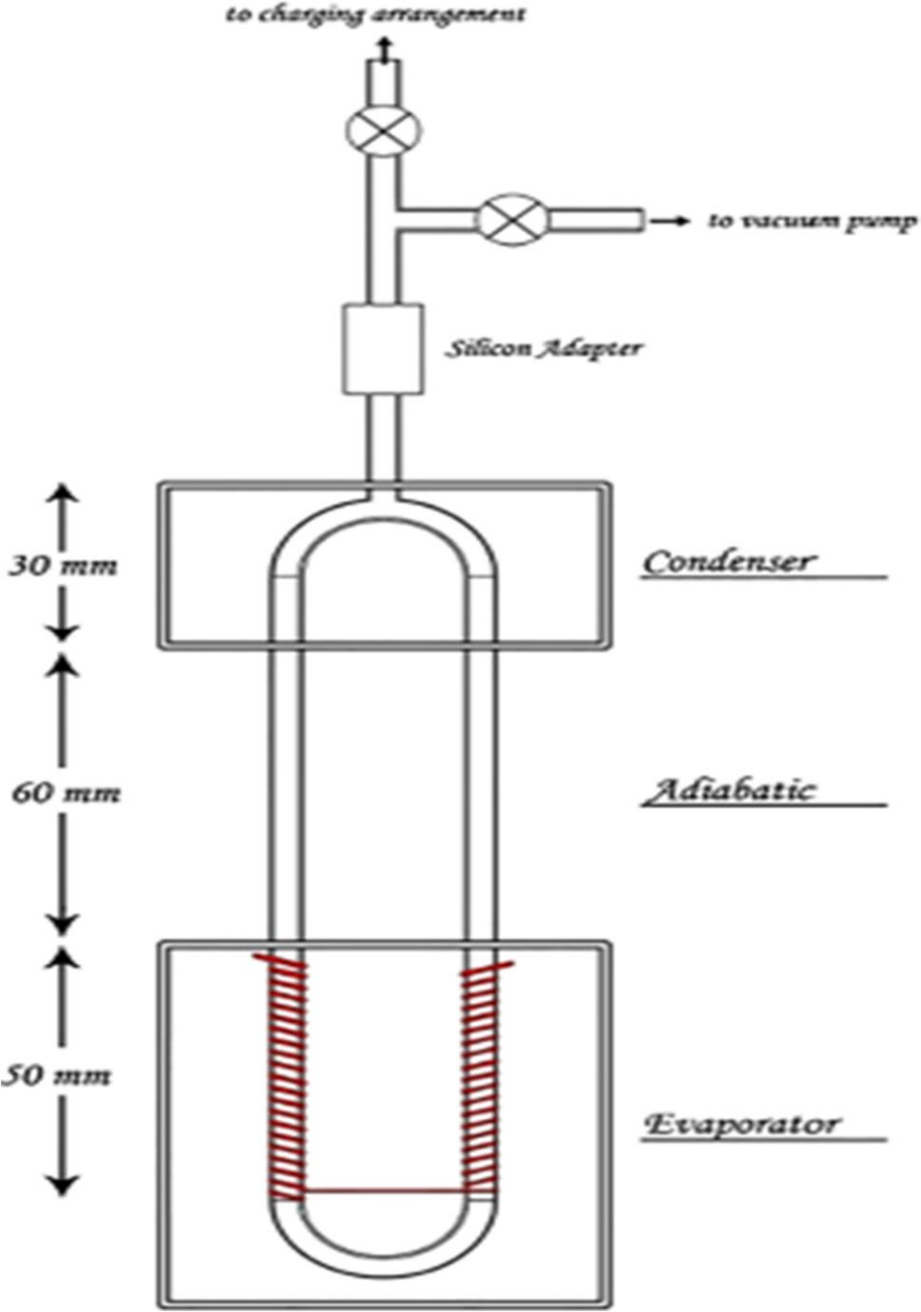
The schematic diagram of PHP (Alizadeh et al., 2018)

**Fig. 24:**
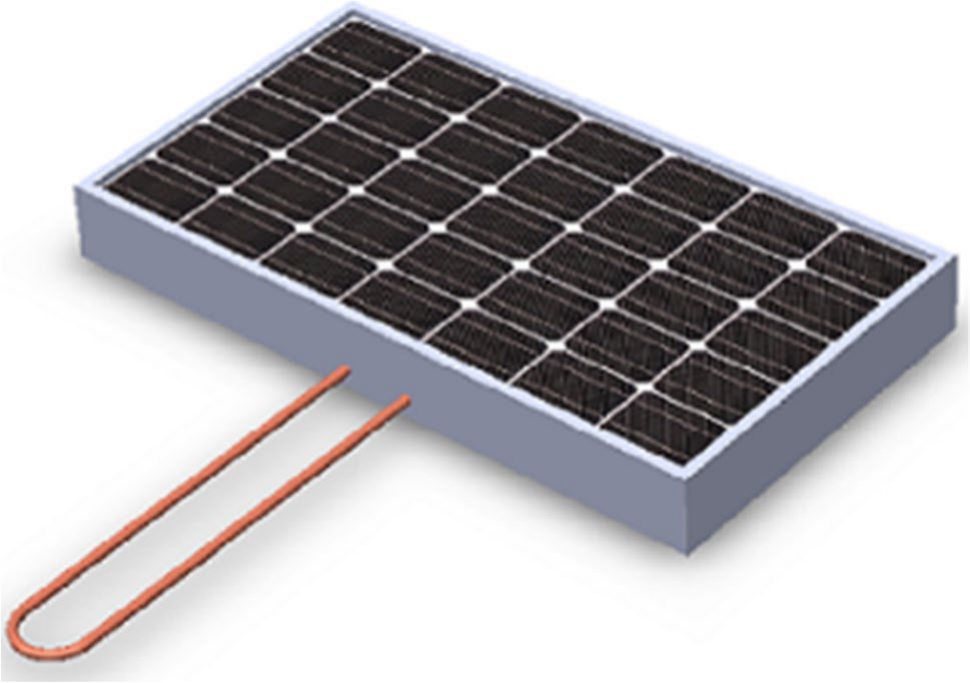
The PV panel integrated with PHP (Alizadeh et al., 2018)

**Fig. 25:**
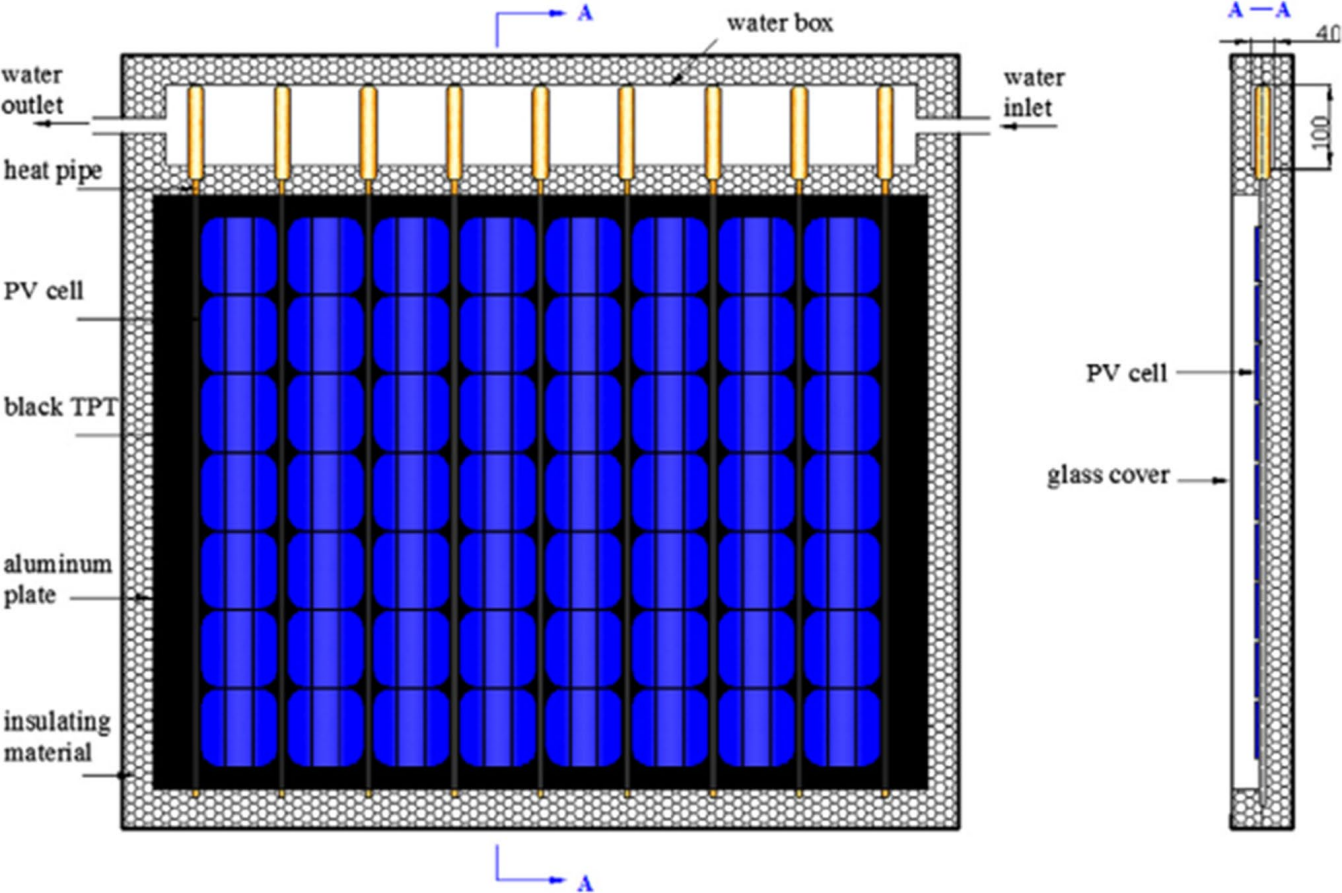
The PV/T system integrated with heat pipe (Gang et al., 2011)

Alizadeh et al. (2018) numerically investigated the cooling of photovoltaic with heat pipe. In their study, they applied a pulsating heat pipe (PHP), as shown in Fig. 23, to the back surface of the photovoltaic to extract the heat as shown in Fig. 24. The results indicated that the PV integrated with PHP has approximately 18% enhancement in electrical efficiency compared with PV without any cooling system. Gang et al. (2011) conducted a practical and theoretical experiment to compare the photovoltaic/thermal system with and without heat pipe. The experiment was investigated to validate the results obtained from the simulation. They integrated the PV/T with heat pipe, as shown in Fig. 25. The experiment results indicated that the thermal and electrical efficiency of PV/T with heat pipe is 41.9% and 9.4%, respectively, while the simulation results agree with the experimental results with the relative error was ± 5%.

**Fig. 26:**
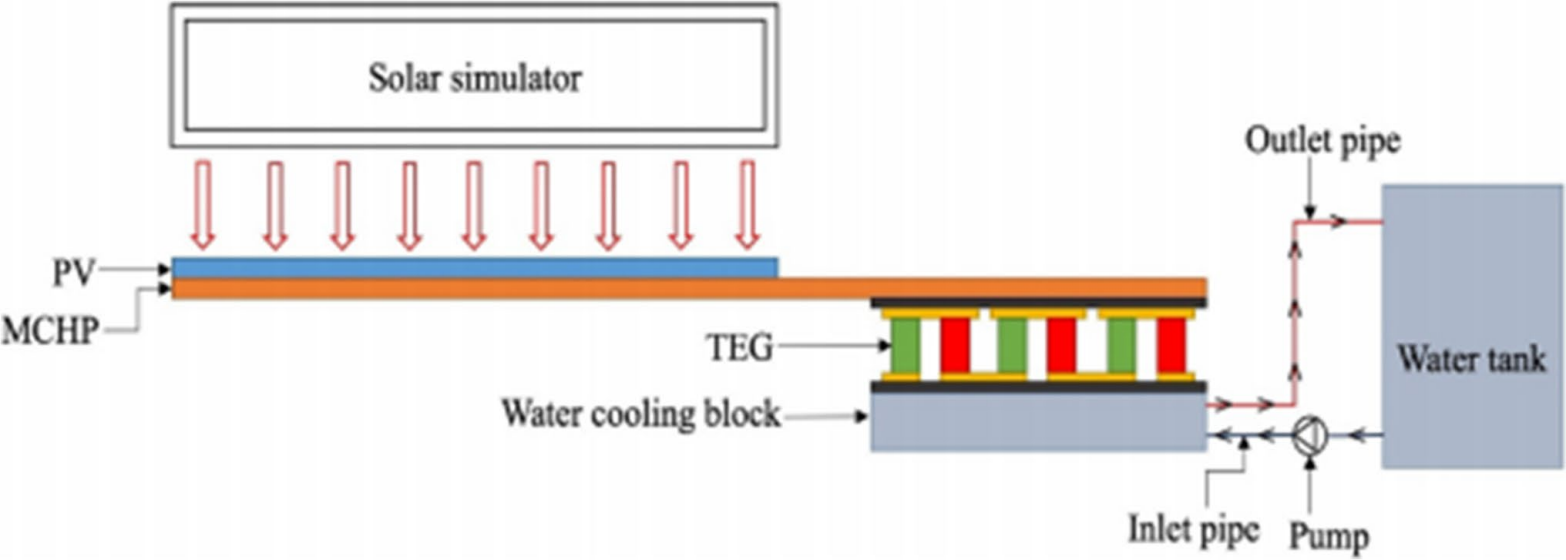
The schematic diagram of Shittu et al. (2020)

Shittu et al. (2020) made a laboratory experiment to study the effect of integrating the photovoltaic system with heat pipe and thermoelectric. The schematic diagram of the experiment is shown in Fig. 26. The experiment was done in the lap under a solar simulator, and the cooling water is used for the thermoelectric generator. The results indicated that the electrical efficiency of the hybrid PV/TEG with heat pipe is increased by 2.1% after 1 h, and there is a temperature reduction in the hybrid system surface temperature by 8.8% compared with the reference PV.

#### Discussion

The literature shows various types of passive cooling mechanisms based on the application of solar PV panels. Immersion cooling, heat pipes, natural air cooling with fins, heat sinks, and improved heat exchanger designs were found to yield uniform temperature in most of the PV installations. Heat pipe cooling with its high heat flux dissipation capability was shown to be effective for PV cooling. Cell temperature was found to be in the range of 32–46 °C with the best-case temperature non-uniformity of 3 °C for concentrated systems. It is a low-cost system based on its passive cooling. Heat sinks and heat spreader are another passive cooling techniques reported in the literature. These systems are capable of reducing the cell temperature to as low as 37 °C even for high concentration levels. The main drawback of these dissipation techniques however is that to keep the cell temperature maintained, heat sink area directly increases with the concentration ratio. As a result, a very large heat sink is required to release heat from concentrated PV systems making them less realistic and attractive for cooling. The viability of passive system economic wise is not suitable since it involves large quantity of material fins/planes.

Table 2 provides a comparison between the different cooling techniques discussed in the “Cooling techniques” section. The comparison is based on the PV surface temperature, initial cost, maintenance cost, heat transfer rate, the power required, and the lifetime of each technique.

**Table 2:** The comparison between cooling techniques

Reference	Cooling technique	PV surface temperature (^o^C)	Initial cost	Maintenance cost	Heat transfer rate	Power required	The lifetime of each technique
(Shahsavar and Ameri, 2010)	Forced air	20:30	High	High	High	High	Lower life
(Odeh and Behnia, 2009)	Forced water	20:30	High	High	High	High	Lower then forced air circulation due to chances of corrosion
(Wu et al., 2018)	PV/T Thermal system	20:30	High	High	High	High	Similar to forced water
(Cuce et al., 2011)	Natural air	50:70	Zero	Zero	Low	Zero	Longer life
(Han et al., 2011)	Natural water	30:45	Low	Low	High	Zero	Less due to corrosion of PV panel
(Gang et al., 2011)	Heat pipe	30:96	High	Low	High	Zero	Longer life

As we mentioned before, using the passive method in cooling the PV solar cells gives slight improvement results, so we resorted to using phase change materials (PCMs) to cool the PV cells. In the next section, we will review the most important researches that dealt with this topic.

## PCM cooling

The phase change materials (PCMs) are used to cool the PV solar cells by absorbing the heat generated in the PV cell until the temperature of the PCM reaches the melting point (sensible heat). Then after that, the PCM begins to absorb another part of the heat generated in the PV until transfer from a solid state to a liquid state (latent heat). As a result of the heat absorbed from the PV cell during the day, the PV cell cooled, and consequently, the power produced from the PV cell increases. Then, during the sun’s absence, the heat absorbed in PCM is transferred to the ambient, and the temperature of the PCM decreases. Then the cooling cycle is restarted the next morning. Once again, many types of research have dealt with this cooling method, which we will review in the following. Among numerous PV thermal regulation techniques, the use of PCM has been suggested as a promising candidate in PV systems due to its significant phase change enthalpy, ease of use, low cost, chemical stability, and applicable application phase transition temperature (Maatallah et al., 2019). Thus, PCM has been widely utilized in various engineering system’ thermal regulation, electronic cooling, solar thermal energy applications, automotive industry, energy conservation of buildings, and waste heat recovery (Yousef and Hassan, 2020, 2019a, 2019b). For thermal regulation of PV systems, when PCM is assimilated at PV back, solar energy dissipated as heat energy is captivated by PCM in the form of latent heat, retaining the PV temperature at an acceptable level and nearly uniform for some period (Yousef et al., 2022).

### PCM selection

**Fig. 27:**
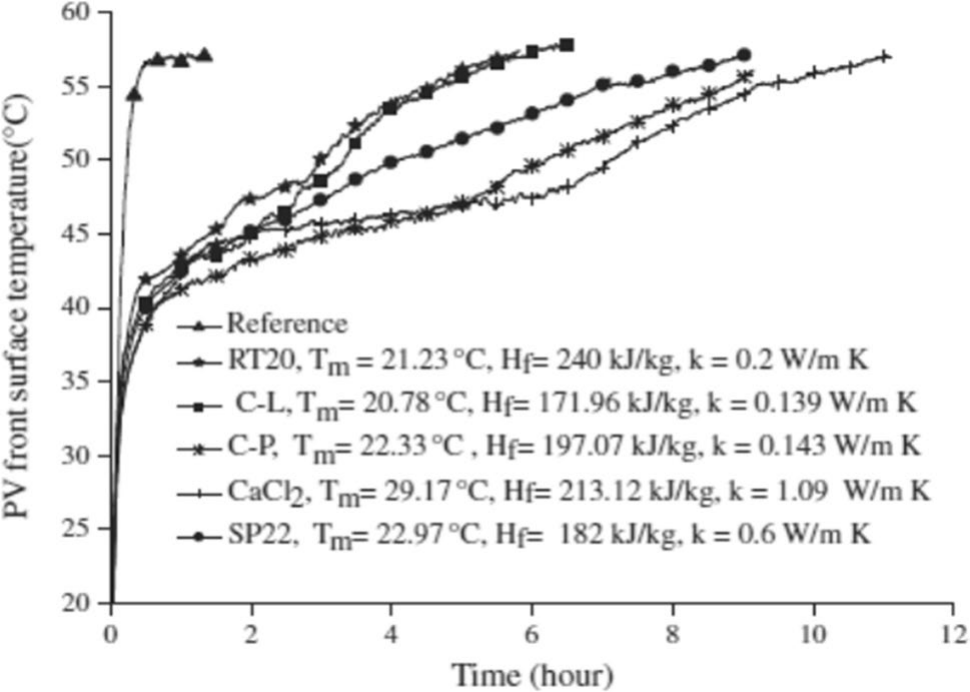
Performance of the five types of PCM on the surface temperature of the PV cell at insolation of 1000 W/m^2^ and the ambient temperature of 20 ± 1 °C (Hasan et al., 2014)

The choice of PCM used to cool the PV solar cell depends on the melting point and the ambient temperature. It is preferable to choose PCM with a melting point less than the ambient temperature by 8–10 °C. The choice of PCM also depends on the properties of the PCM itself, which includes the thermal conductivity and the amount of the latent heat of fusion (Hasan et al., 2014). Hasan et al. (2014) conducted an experiment to compare the use of five different types of PCMs, both organic and inorganic with the different melting points with a range from 21 to 30 °C, and the amount of latent heat with a range from 170 to 240 kJ/kg. It became clear from the results that some materials cannot expel the heat during the discharging process due to the low freezing temperature, while others are characterized by better performance during the charging and discharging process. Figure 27 shows the performance of the five types of PCM on the cell surface temperature. Hasan et al. (2014) summarized the required properties of PCM and the reason for each property in Table 3.

**Table 3:** Properties of PCM and the reason for each property (Hasan et al., 2014)

Properties	Requirement	Reason of requirement
Thermal	• High latent heat • High heat capacity • Good thermal conductivity • Reversible phase change • Fixed melting point	• Maximum heat absorption • Minimum sensible heating • Efficient heat removal • Diurnal response • Consistent behavior
Physical	• Congruent meting • Low volume expansion • High density	• Minimum thermal gradient • No overdesign • Low containment requirement
Kinetic	• No super cooling • Good crystallization rate	• Easy to freeze • Faster solidification
Chemical	• Chemical stability • Non-corrosive • Non-flammable • Non-explosive • Non-toxic	• Long life • Long container live • Comply with building safety code • Environment friendly
Economic	• Abundant • Cheap and cost-effective	• Market competitiveness • Economic viability and market Penetration
Environmental	• Recyclable/reusable • Odor free	• Ease to dispose of comfortable to apply in dwelling environment

### Pure PCM

Hasan et al. (2017) conducted a practical experiment in the UAE covering the four seasons of the year. They used paraffin wax as a PCM with a melting point (38 to 43 °C) on the back surface of the cell. The results indicate that paraffin cannot fully solidify during the summer due to the high temperature at night and is also unable to completely melt during winter due to the low temperature at daytime. The best results were obtained during the spring and autumn seasons, while the amount of power produced from the cell increased by 5% over the year. In another investigation conducted by Du et al. (2016), they compared the performance of PV panels integrated with two finned PCMs, namely capricpalmitic acid (PCM1) and CaCl_2_·H_2_O (PCM2) for warmer and cooler climates of Vehari and Dublin, respectively. In Dublin, results revealed that maximum cell temperature declined from 48 to 42 °C and 39 °C by using PCM1 and PCM2, leading to about 4% and 11.2% enhancement in electricity yield, respectively, whereas in Vehari, it brought down from 62 to 50 °C and 41 °C with PCM1 and PCM2, resulting in about 5.2% and 13% enhancement in electricity yield, respectively. Overall, it was reported that employing the proposed system could be economically viable in Vehari than in Dublin. Hasan et al. (2016) studied the integration of PCM (RT42) with a BIPV panel with vertical fins fixed at its rear under hot climate conditions. The findings reported that the proposed cooling scheme contributed to PV-temperature reduction by 21.8 °C for conventional PV, causing an improvement in electrical power by 7.3%.

Stropnik and Stritih (2016) performed an experiment to cool the PV solar cell using phase change material (RT28) throughout the year. The results proved an improvement in the power output from the modified cell (PV + PCM) by 4.3–8.7% and also an improvement in the electrical efficiency of the PV cell by 0.5–1% compared to the conventional cell. It also became clear that the annual increase in energy was 7.3% and in efficiency was 0.8%, compared to the conventional cell. The feasibility of utilizing petroleum jelly as a PCM inserted in metal pipes and fixed underneath the PV for its thermal management was tested by Indartono et al. (2016). The results revealed that the electrical efficiency of a traditional PV system and modified PV-PCM system was 8.2% and 10.3%, respectively. Nižetić et al. (2018) examined through numerical investigation the potential of using pork fat as PCM to passively cool the PV panels by comparing its annual performance with traditional organic PCM under Croatian climate conditions. The insignificant electrical yield was observed due to the highest close physical properties of the two considered PCMs, i.e., latent heat of fusion and melting temperature range. Although pork fat was eco-friendly and had lesser investment, it was not economically worthwhile because of its deterioration in thermo-physical characteristics on a long-lasting basis.

**Fig. 28:**
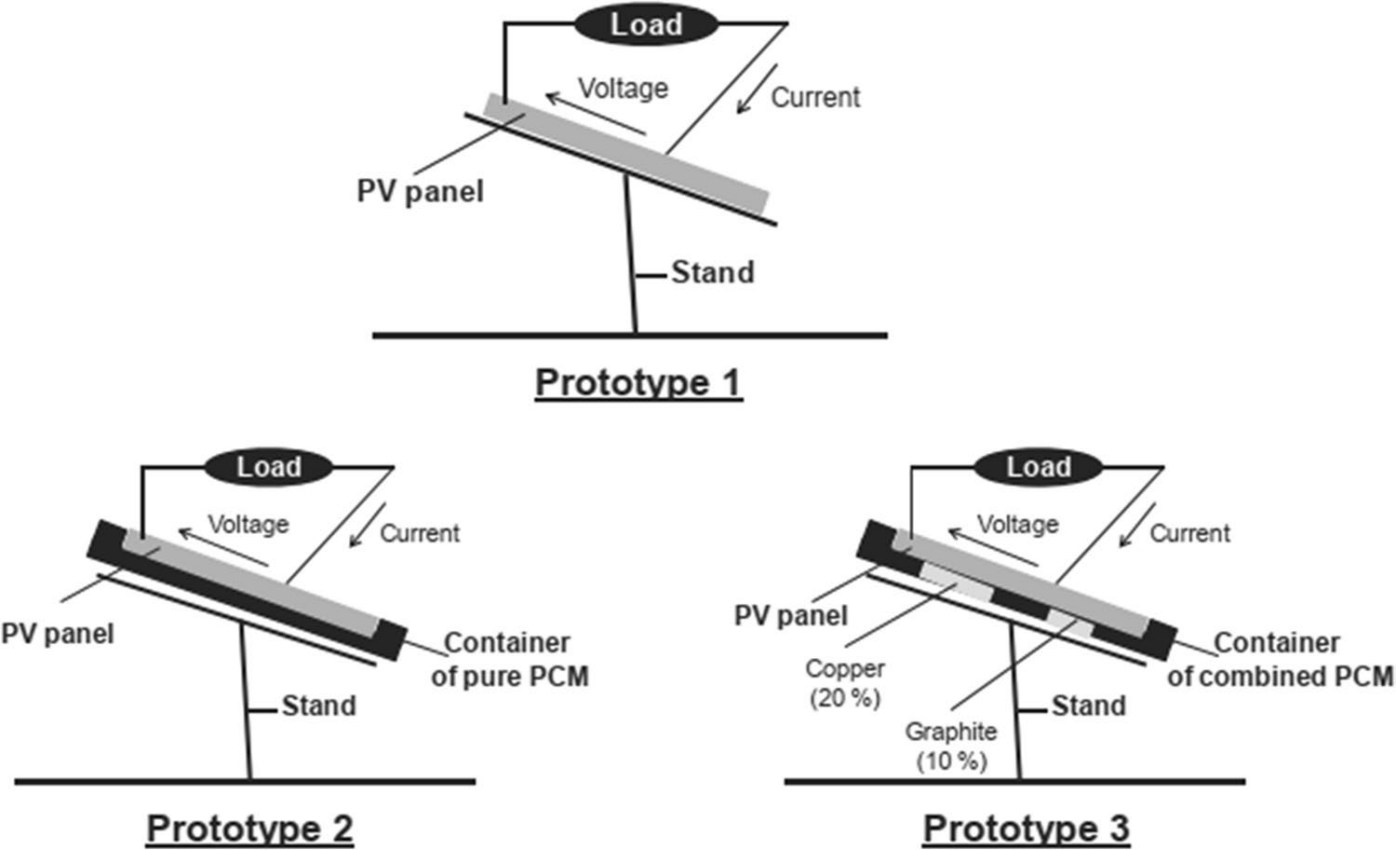
Schematic of Hachem et al. (2017) experiment

Another simulation work was completed by Groulx and Biwole (2014) on examination of PV-PCM system by changing tilt angle from 0 to 90° from the vertical. At an inclination angle of 0°, heat transfer was completely by free convection, whereas at an inclination angle of 90°, heat transfer was governed by pure conduction. Around 23 °C, further temperature dropping was perceived in vertical location than in horizontal location. Kant et al. (2016) examined the heat transfer mechanisms of the PV-PCM system and its effects on power output through numerical simulations. The findings indicated that the maximum cell temperature declined by 3 °C and 6 °C with conduction and convection heat transfer modes, respectively, indicating convection governance. Also, it was reported that increasing the angle of inclination and wind velocity results in lower cell temperature of the PV-PCM system, thus enhancing its efficiency. Hachem et al. (2017) experimentally cooled the PV solar cells using phase change materials. They used in the experiment three PV cells, the first was without any modifications, the second was modified by adding a box that holds one type of PCM (white petroleum jelly), and the third was modified by adding a box that holds three types of PCM (white petroleum jelly, copper, and graphite) as shown in Fig. 28. The results showed an increase in the electrical efficiency of the second PV cell by 3% and an increase in the electrical efficiency of the third PV cell by 5.5%.

**Fig. 29:**
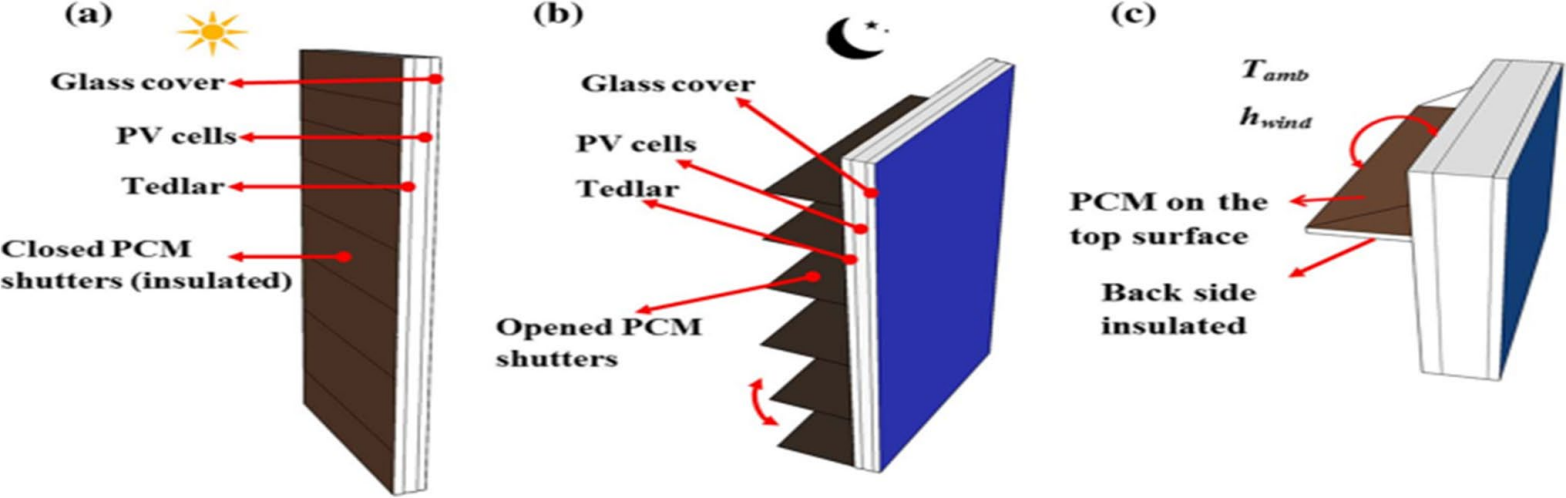
PV solar cell with rotatable shutters for Waqas and Ji (2017) experiment

Waqas and Ji (2017) did an experiment to cool the PV solar cells using PCM. They indicated in their experiment that the PCM is inside rotatable shutters in order to adhere to the surface of the PV cell during the day and absorb heat from it; at night, the rotatable shutters move away from the back surface of the PV cell, so it is exposed to the ambient in order to lose the heat gained, as shown in Fig. 29. The results showed that the maximum increase in electrical efficiency for summer was 9% when using PCM with a melting point of 35 °C. For winter, the maximum increase in electrical efficiency was 2.2% when using PCM with melting point 30 °C, so the use of PCM has a better enhancement in summer seasons than in winter seasons.

**Fig. 30:**
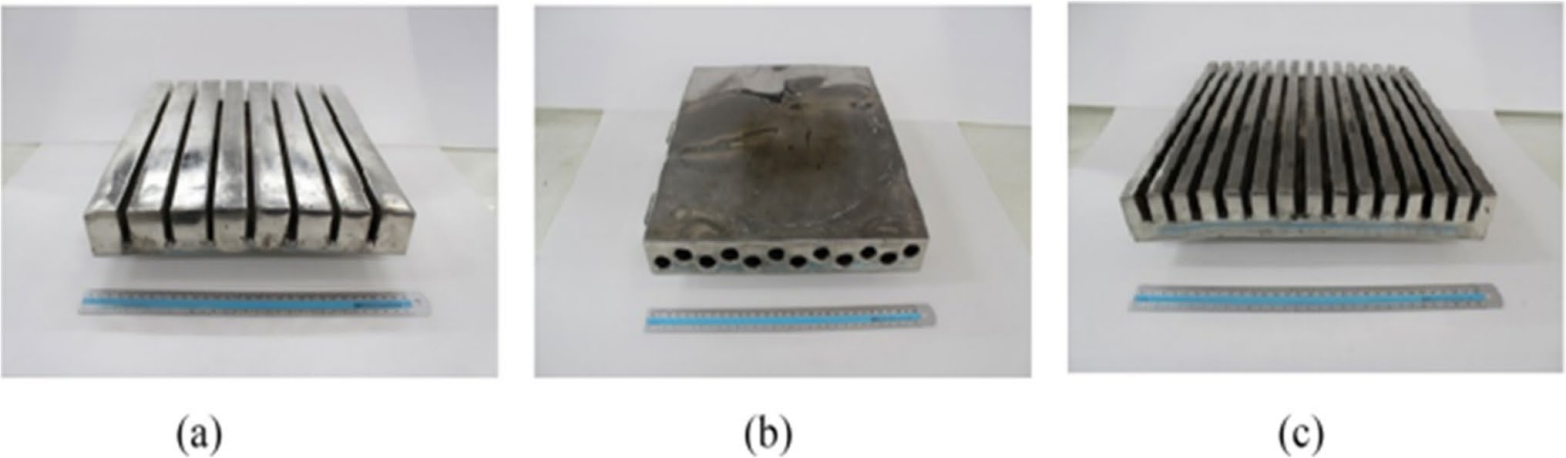
PCM heat sinks: **a** grooved container, **b** tubed container, **c** finned container (Wongwuttanasatian et al., 2020)

Thermal regulation of an air-based PV collector using palm wax as storage material was experimentally examined by Wongwuttanasatian et al. (2020). Comparative performance evaluation of three diverse configurations of PCM containers as shown in Fig. 30, i.e., grooved, tubed, and finned, was conducted. The finned container exhibited superior cooling performance in contrast to the other container’s configurations, reducing the temperature of the solar cell by roughly 6.1 °C and thereby achieving a 5.3% increment in electrical efficiency compared to the uncooled PV module.

In a house-regulated experiment, thermal management of low concentration BICPV system using RT42 PCM was implemented by Sharma et al. (2015). The use of PCM contributed to the highest reduction of 5.2 °C in the PV temperature, thus leading to a 15.9% increment in electrical efficiency in contrast with the uncooled BICPV system. In another study conducted by the same authors (Sharma et al., 2016), under indoor experiments, using RT42 PCM at 1000 W/${\mathrm{m}}^{2}$ solar intensity aids in improving the electrical efficiency of BICPV by 7.7%. The transient performance of a PV-PCM system considering the impact of weather conditions was theoretically scrutinized by Khanna et al. (2018). The simulation results indicated that PCM incorporation with PV modules has more potential for a climate with fewer ambient temperature fluctuations. Due to using PCM as a passive cooling scheme, it was observed that a 9.8% enhancement in electricity yield for the weather with little fluctuations in environmental temperature and only a 6.8% for weather having big fluctuations. The electrical performance enhancement of PV system using two approaches of forced air cooling and PCM was examined by Khanna et al. (2019). It was found for the PCM layer of depth 5 cm decreasing the angle of wind from 75° to 0°, prolonging the electrical enhancement duration from 7.1 to 8.5 h, and reducing the power output from 17.4 to 13.5 W/m^2^. Furthermore, it was reported declining the wind speed from 6 to 0.2 m/s, shortening the electrical enhancement duration from 9.2 to 6.5 h, and enhancing the power output from 11.7 to 22.6 W/m^2^.

#### Discussion

PCM is quite effective in absorbing PV-panel extra heat which is not converted into electrical energy. Latent heat of PCM helps in lowering panel temperature, bringing it closer to 25 °C and maintaining it almost constant during peak sunny hours. Maximum temperature reduction of about 35 °C is brought using PCM by Hasan et al. (2016), Stropnik and Stritih (2016), and Waqas and Ji (2017). Most of the work is done in summer conditions due to high ambient temperature and solar intensity. In summer, PCM mostly used is RT42 due to high PV-surface temperatures, while PCM with melting point in range of 25–30 °C is mostly used in winter conditions as less reduction in PV temperature is required (Ali, 2020). PV-efficiency enhancement up to 16% can be achieved by PCM. The literatures show that the efficiency enhancement in summer is higher than that in winter, so PCM incorporation is more effective in summer (Hasan et al., 2017). The reason behind this is higher heat absorption in summer by PCM in the form of its latent heat. It is found that significant increase in PV power output can be achieved with PCM as compared to without PCM. Max. enhancement is 23% by Stropnik and Stritih (2016) who used RT28 HC which brought panel temperature close to 25 °C, where PV shows maximum power output.

### Integrated PCM with PV/T thermal system

**Fig. 31:**
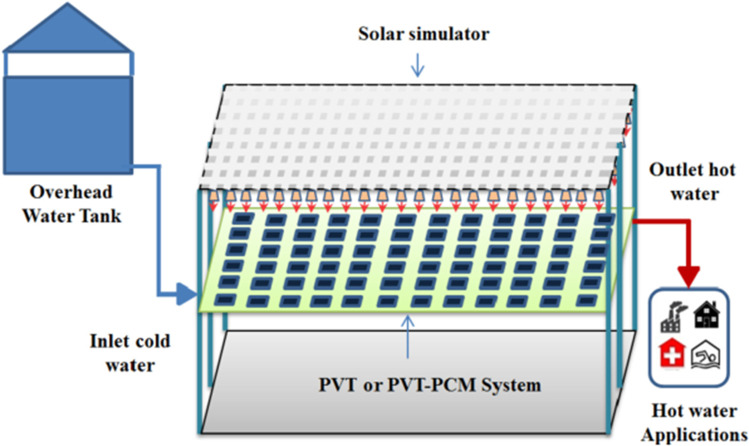
The design of the second or third PV cell for Fayaz et al. (2019)

Fayaz et al. (2019) conducted a practical experiment and theoretical analysis to cool the solar PV cell and used three cells, the first without any additives, the second (PV/T), and the third (PVT/PCM). Figure 31 shows the design of the second or third PV cell. As this system is used to cool the cells, it is also used to obtain hot water. It became clear from the theoretical results that the electrical efficiency of the three cells reaches 11.35%, 12.4%, and 12.75% for PV, PV/T, and PVT/PCM, respectively. From the practical results, the electrical efficiency of the three cells reaches 11.15%, 12.28%, and 12.59%, respectively. It also became clear that the overall efficiency of PV/T and PVT/PCM reaches 89.6% and 83.95%, respectively, for practical cases. Browne et al. (2016) inspected the effects of using palmitic acid/capric mixture as PCM material on the electrical performance of PV/T system through outdoor experiments. Water circulation through the heat exchanger embedded in the PV/T system was done using a thermosyphon closed-loop flow approach. The results exhibited that utilization of PCM with PV/T system contributed to more than 5 °C reductions in cell temperature compared to that of PV/T system in the absence of PCM.

Gaur et al. (2017) constructed a mathematical model for comparing cooling cells using PV/T and PV/T with PCM. The results showed that the electrical efficiency of PV/T with PCM is higher than the electrical efficiency of PV/T. Still, the thermal and the overall efficiency of PV/T with PCM is lower than the thermal and the overall efficiency of PV/T.

**Fig. 32:**
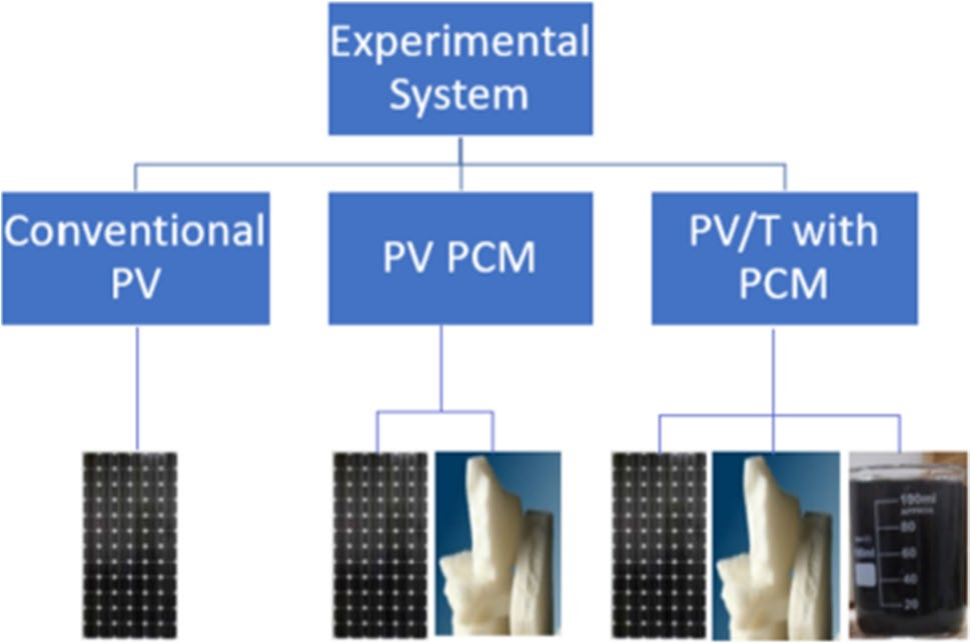
Modules of PV for Hassan et al. (2020)

Hassan et al. (2020) constructed an experimental model to cool the PV solar cells using four cells, the first without any additives, the second (PV/PCM), the third (PVT/PCM), and the fourth similar to the third, but they added nanofluid (graphene) to the water used to enhance the heat transfer inside it as shown in Fig. 32. The results proved that the electrical efficiency increased by 23.9%, 22.7%, and 9.1% for the fourth, third, and second modules compared to the first module. The results also showed an increase in thermal efficiency by 17.5% due to using the nanofluid with water. Yang et al. (2018) experimentally compared the performance of water-based PV/T systems with and without capric acid as PCM. Incorporating PCM with PV/T system boosted the thermal efficiency, electrical efficiency, and overall performance by 20.54%, 16.9%, and 20.24%, respectively. Hossain et al. (2019) conducted a practical experiment to cool the PV solar cell by using two cells, the first is conventional without any additives, and the second is (PVT/PCM). The results proved that the maximum electrical efficiency of the second cell is 14.42%, with an increase of 4.72% over the first cell, and the thermal efficiency of the second cell reached 87.72%.

Effects of water flow rate and inlet temperature on PVT/PCM performance were determined through numerical simulation results by Zhou et al. (2017). Different water flowrates (0.25, 0.5, 1, 5, 10 kg/s) and inlet temperature values (10, 15, 20, 25 °C) were investigated. Increasing water flowrate and decreasing its inlet temperature resulted in cell temperature reduction and PV-efficiency enhancement. Moreover, effect of water inlet temperature had more impact than its flowrate on PVT performance.

#### Discussion

In this method, we see that combination of passive and active cooling techniques is more effective than passive cooling alone. Max. reduction in PV temperature (i.e., about 50 °C) is achieved by Zhou et al. (2017). It also shows that flow rate of water is one of the important parameters in designing such PV systems. Reason is absorption of PV extra heat by PCM (i.e., passive cooling) followed by water (i.e., active cooling) flowing at high flow rate through tubes inside PCM. In this way, the combination of PCM and PV/T caused an increase for the electrical efficiency and thermal efficiency, according to Hossain et al. (2019), Yang et al. (2018), and Hassan et al. (2020), while Gaur et al. (2017) found the decreasing trend in the thermal efficiency. As efficiency of this system increases due to its high capacity of excess heat absorption, power output also increases accordingly making it very effective PV system.

### PCM with additives

Despite the several promising features of PCMs in terms of high-energy density with small storage volume, their poor thermal conductivity restrains the effectiveness of the heat transfer process during absorbing and releasing processes (Yousef et al., 2019; Yousef and Hassan, 2019c). Numerous heat transfer enhancement techniques were examined by many scholars to tackle this shortcoming, improve the thermal conductivity, and improve the performance of PCM in the process of cooling PV solar cells, such as using extended surfaces (fins), dispersing nanoparticles within the PCM, adding expanded graphite, encapsulation of PCMs, embedding heat pipes, using multiple PCMs, and using foam structures. Such methods are based on augmenting the contact area between the PCM and the heat source (Xu et al., 2018).

#### PCM with fins

**Fig. 33:**
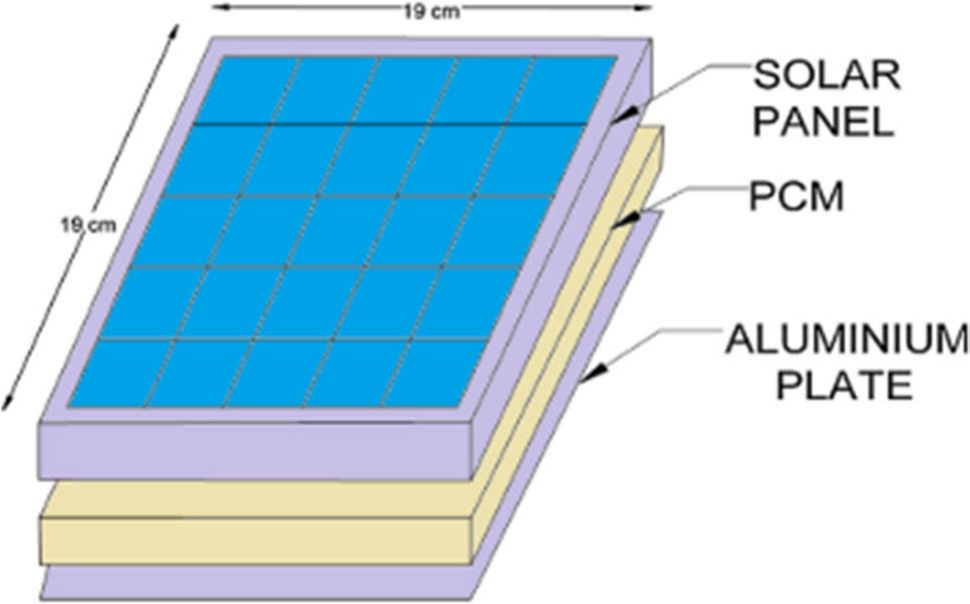
Three-dimensional sketched of PV/PCM with aluminum sheet for M. et al. (2019)

The effectiveness of using fins inside PCM (RT27) on the performance of PV panels under outdoor conditions was reported by Tan et al. (2017). Experiments are conducted for different numbers of fins, i.e., finless, 3, 6, and 12 fins. The findings revealed that PV-PCM with 12 fins demonstrated the best performance, achieving a maximum reduction of 16 °C in PV temperature, with a 5.9% improvement in PV efficiency. In indoor experiments conducted by Kumar et al. (2020), a comparative performance assessment of three PV systems, namely typical PV (case 1), PV with PCM (case 2), and PV with PCM filled in the container having external fins (case 3), was performed under warm weather conditions. A reduction of 11.3 °C and 22 °C in PV temperature was reported for cases 2 and 3, respectively, along with an enhancement of 4.25% and 6.48% in the electrical performance. Qasim et al. (2020) investigated the performance enhancement of PV systems through extensive experiments using two approaches, i.e., incorporating finned PCM with PV with using a different number of fins and using two PCMs having various melting temperatures, disjointed by finned plate. The usage of 2-fin, 5-fin, 8-fin, and 11-fin assisted in lowering the maximum PV temperature by 23.8 °C, 24.2 °C, 25.1 °C, and 26.6 °C, enhancing the electrical efficiency from 10.1 to 11.2%, 11.7%, 11.9%, and 12.1% respectively. Furthermore, it was reported that using a single PCM arrangement was more superior to two PCM arrangements. Using single and two PCMs, arrangements lowered the temperature of the solar cell by 9.6 °C and 7.8 °C, leading to performance enhancement of 13.2% and 8.7%, respectively, as compared to conventional PV. Huang et al. (2006) also studied the placing of fins inside the PCM to improve its thermal conductivity; M. et al. (2019) used an aluminum plate with PCM to work as thermal conductivity enhancers, as shown in Fig. 33. The results showed an increase in the average electrical efficiency of the PV solar cell = 2%.

##### Discussion

In the using of finned PCM, PV-temperature reduction values mostly range between 5 and 20 °C, while max. value is about 26.6 °C obtained by Qasim et al. (2020) who used RT27 having Tm = 26.6 °C, very close to the ideal PV temperature of 25 °C where PV module performs at its best. Effect of fin insertion inside PCM reduced PV-surface temperature due to the fact that presence of fins increased conduction heat transfer rate within PCM, which is very much required as thermal conductivity of PCM is very low. This increased transfer of excess heat from PV surface to PCM followed by enhanced PV performance. Fins also prolonged temperature regulation period during peak hours and brought uniformity in PV-surface temperature as well. Reason is high heat absorbing capacity of PCM along with increased conduction rate due to fins inserted inside PCM. Fins enhanced temperature regulation period during peak hours, thereby hindering power output decline.

#### Composite PCM

##### PCM with nanoparicles

**Fig. 34:**
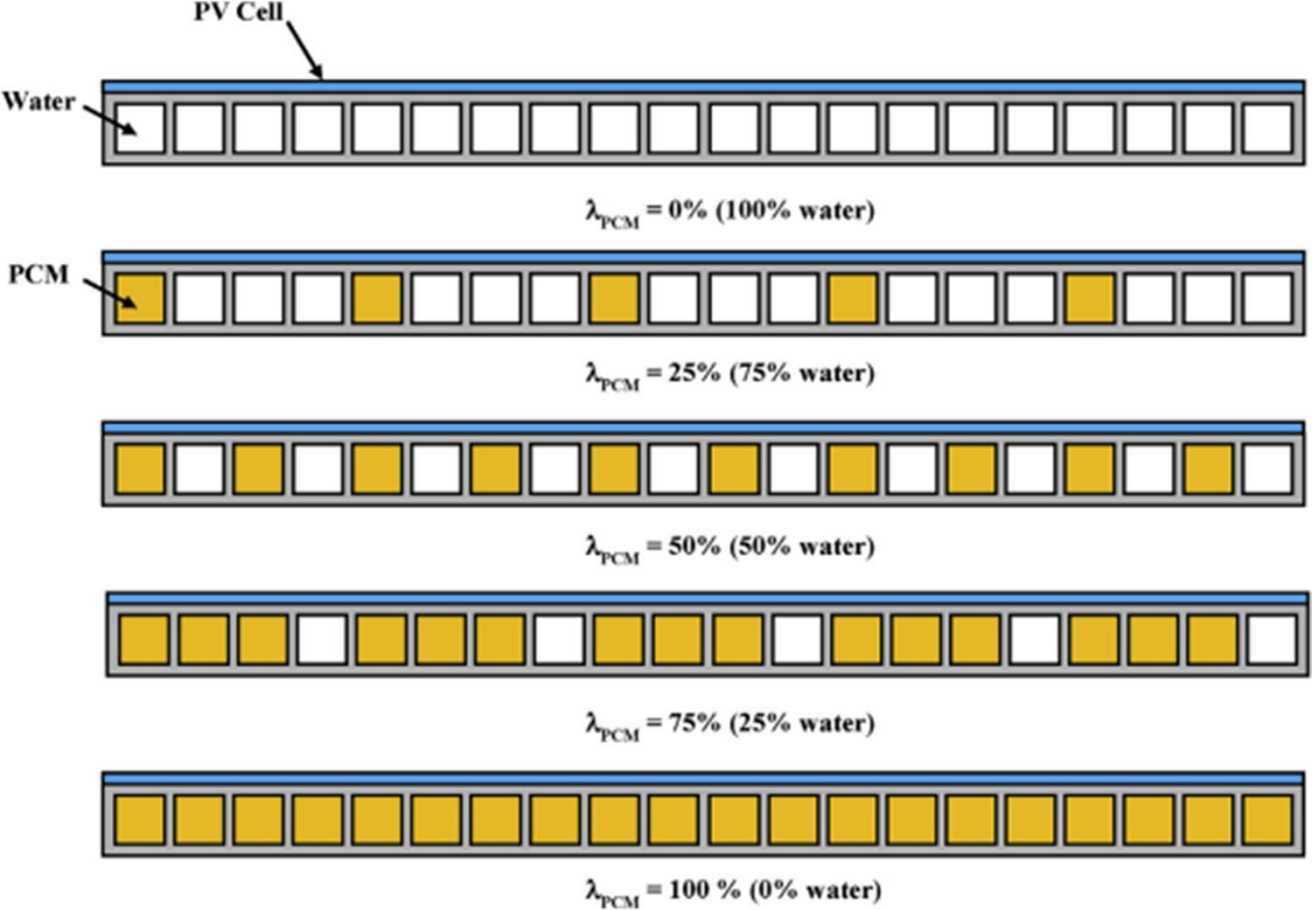
PCM and water occupation for Salem et al. (2019)

Many studies have been conducted on mixing nanoparticles with PCM to improve the thermal conductivity of the PCM. Nada et al. (2018) conducted a practical experiment using three PV cells, the first without any additives, the second by adding PCM only, and the third by adding PCM mixed with A ${l}_{2}{\mathrm{O}}_{3}$ nanoparticles, and it became clear from the results that the electrical efficiency increased by 5.7% and 13.2% for the second and third PV cells, respectively. Al-Waeli et al. (2017) conducted a practical experiment to improve the performance of the PV/T by adding nanofluid to water and adding nano-SiC particles to PCM. The results showed an increase in electrical efficiency from 7.1 to 13.7%, and the thermal efficiency reached 72%. Siahkamari et al. (2019) also added CuO nanoparticles to PCM to improve performance, and the results showed an improvement in the power output by 6.5%. Salem et al. (2019) conducted an experiment to cool the PV cell by placing tubes of equal cross-sectional area in the back of the PV cell, passing water in some tubes, and placing PCM with Al_2_O_3_ nanoparticles in other tubes in different percentages, as shown in Fig. 34. The results proved the best electrical and thermal efficiency when using 25% PCM + nano and the rest (75%) water.

##### PCM with porous metal

**Fig. 35:**
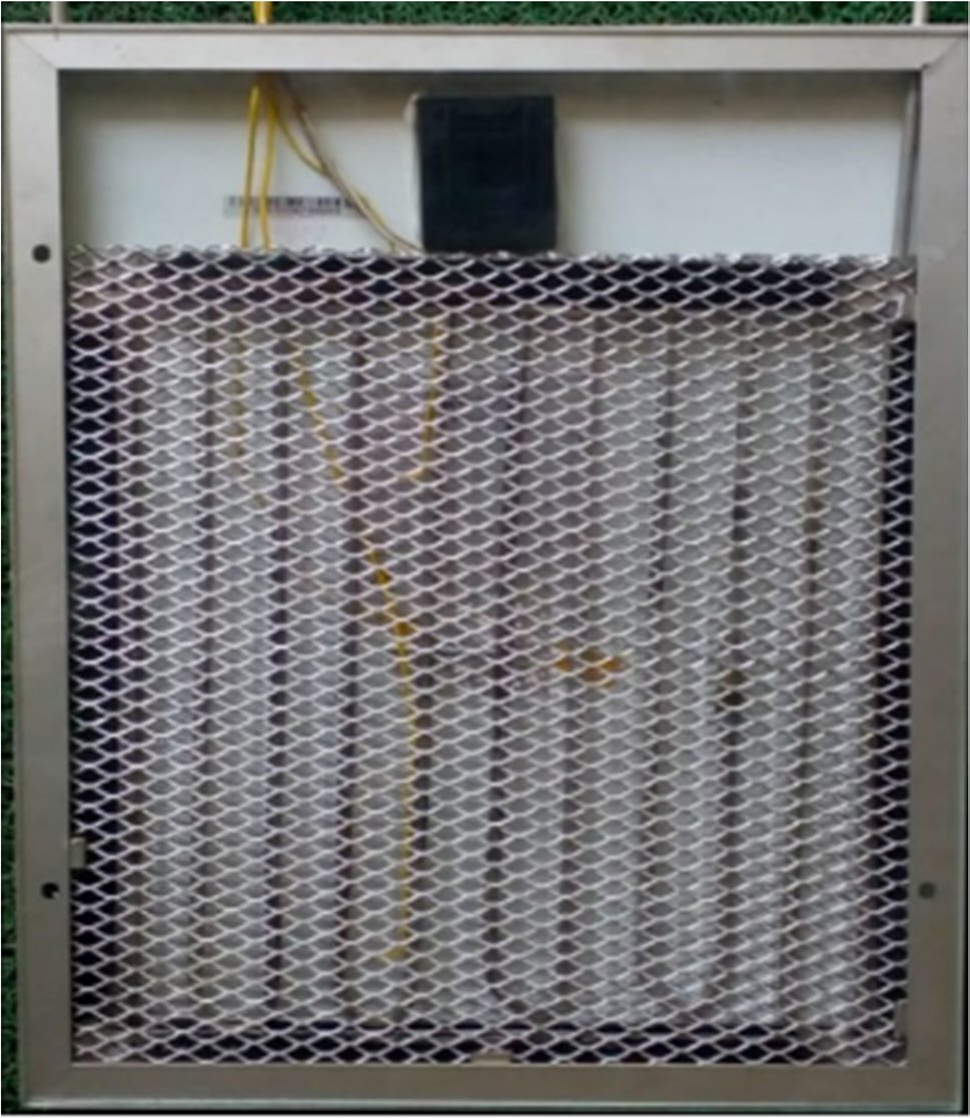
PV/T with an aluminum matrix for Shastry and Arunachala (2020)

Among all, the embedding of porous metal foams in PCMs has been ascertained as the most thermally effective technique due to its various attractive features such as large surface area per unit of volume, lightweight with high stiffness, high thermal conductivity, and high fluid permeability as compared to pure PCM. The effectiveness of metal foams is governed by different parameters such as the metal material, porosity, and pore density. Metal foams boost the rate of heat transfer by augmenting the exposure area between the heat source (absorber plate) and the working fluid (PCM), providing enhanced heat transfer capability with a slight penalty on the thermal storage capacity (Hajjar et al., 2020). As a consequence, the high potential of impregnating PCM with porous metal foams has already been reported in many experimental and numerical investigations (Chen et al., 2021), such as Zheng et al. (2018), Zhao et al. (2010), Ghahremannezhad et al. (2020), Dinesh and Bhattacharya (2020), and Mancin et al. (2015). They concluded that employing porous metal foams could result in better temperature uniformity and considerably decrease the melting time of PCM. Also, another study reported that the heat transfer of PCM-porous composite was ten times as greater than the pure PCM (Duan and Li, 2021). Shastry and Arunachala (2020) did an experiment to improve the performance of the PV/T. They inserted an aluminum matrix in the PCM to improve its thermal conductivity and increase its melting and solidification rate. The results proved that the conversion efficiency of the PV cell with aluminum matrix increased by 8%, while it increased by 3.5% in the case of using PCM only. Figure 35 shows the PV cell after adding the matrix to it.

Mousavi et al. (2018) numerically studied the performance of a composite of PCM/copper foam incorporated with a water-based PV/T system based on thermal, electrical, and exergy approaches. Five different PCMs were considered: sodium phosphate salt, capric/palmitic acid, and paraffin C15, C18, and C22. It was reported that the maximum thermal efficiency of 84% was obtained using paraffin C22 as PCM storage material. Also, the results indicated that incorporating copper foam with a porosity of 0.8 into the PCM enhanced the thermal and electrical efficiency by 1.1% and 2.23%, respectively. Furthermore, the exergy of the modified system was the highest (around 16.7%).

Abdulmunem et al. (2020) conducted an experiment for cooling the PV solar cells and used three cells, the first without any additives, the second with PCM only, and the third with PCM and copper foam matrix. The results proved that the electrical efficiency of the third cell increased by 5.68% compared to the first cell, while the efficiency of the second cell increased by 1.97% compared to the first cell. Vaziri Rad et al. (2021) explained through experiments conducted under hot and cold climate conditions the thermal, electrical, and exergetic performance of water-based PV/T incorporated with PCM (salt hydrate) and aluminum shavings which act as a porous medium. A reduction of 24 °C in PV temperature was observed due to using the proposed system, resulting in an increment in the electrical performance by 2.5%, compared to reference PV. The usage of aluminum shavings aided in accelerating the melting time of PCM by a maximum of 25%. Also, it was reported that the proposed system achieved a maximum exergy efficiency enhancement of 4.43% against the reference PV.

However, few investigations have been completed to enhance the thermal response of PCM through using metal foams for the thermal management of PV modules. Klemm et al. (2017) examined the influence of integrating metal fibers to PCM (RT54) for passive PV cooling through numerical investigations. Presence of metallic fibers in the PCM layer having 50 mm thickness assisted in dropping PV temperature by a maximum of 21 °C, while their porosity had little influence on performance. Duan (2021) numerically investigated the effectiveness of PCM incorporated with metal foam porous for passive CPV cooling at CR of 20. Also, the influence of varying porosity and height of metal foam was considered. The usage of a PCM-porous system as a heat sink significantly improves the cooling impact of CPV as opposed to the normal PCM. It was reported that decreasing the porosity enhances the electrical performance of the CPV system while the period time which could retain the CPV in a uniform temperature shortened. Recently, Ahmadi et al. (2021) have conducted indoor experiments to examine the performance of PV panels passively and actively cooled by carbon foam embedded in PCM and passing water underneath PV, respectively, under a broad range of solar irradiance. The findings indicate that PCM composite as a passive cooling approach declined the PV temperature by 6.9%, enhancing the PV electrical efficiency by 13.9%. Furthermore, it was reported that the thermal efficiency of the proposed system with active cooling enhanced up to 81.6%.

##### Discussion

Through the previous studies, this method gives the best results in improving electrical efficiency and output power of the PV, so this method is considered the best method among all other cooling methods. The reason is that although nanoparticles and porous metal addition in PCM decreased heat absorbing capacity very slightly, it enhanced its thermal conductivity which increased heat conduction, thereby absorbing panel extra heat and so hindered its power decline trend. Overall, PCM with high thermal conductivity, i.e., compound PCM, is more effective in PV cooling in terms of temperature drop and slower temperature rise after PCM melting. Table 4 shows some important results in the literature that dealt with the cooling of PV cells using composite PCM.

**Table 4:** Some investigations in the literature dealt with the cooling of PV cells using composite PCM

Reference	Included medium	PCM type	System	Key findings
Salem et al. (Salem et al., 2019)	Nanoparticles Al_2_O_3_	Calcium chloride hexahydrate; CaC_l2_H_12_O_6_	PV/T	PV cell temperature declined by 14.5 °C Electrical efficiency enhancement was 22.7% Thermal efficiency enhanced by 78% The maximum percentage increase in the exergy efficiency was 52.3%
Nada et al. (Nada et al., 2018)	Nanoparticles Al_2_O_3_	Paraffin (RT56)	PV	PV cell temperature declined by 10.6 °C Electrical efficiency enhanced by 12.1%
Al-Waeli et al.(Al-Waeli et al., 2017)	Nanoparticles SiC	Paraffin Wax (RT49)	PV/T	PV temperature dropped by a maximum of 17 °C The electrical efficiency increase from 7.1 to 13.7% The maximum thermal energy gained was 69%
Hassan et al. (Hassan et al., 2020)	Nanoparticles graphene	RT-35HC	PV/T	PV temperature dropped by 23.9 °C Electrical efficiency enhanced by 23.9% Overall efficiency enhanced by 12%
Siahkamari et al. (Siahkamari et al., 2019)	Nanoparticles CuO	Sheep fat	PV/T	PV temperature declined by 14.2 °C Overall output power was enhanced by 25.4%
Rajvikram et al. (Rajvikram et al., 2019)	Aluminum sheet	OM-29	PV	PV temperature declined by 10.35 °C Conversion efficiency has been increased by 24.4%
Mousavi et al. (Mousavi et al., 2018)	Copper metal foam	Paraffin	PV/T	Maximum thermal efficiency was 83% Electrical efficiency enhanced by 4.3% Exergy efficiency enhanced by 1.2%
Hossain et al. (Hossain et al., 2019)	Aluminum foil packets	Lauric acid	PV/T	Electrical efficiency enhanced by 2.1% Maximum thermal efficiency was 87.8% Average exergy efficiency was 12.28%
Shastry and Arunachala (Shastry and Arunachala, 2020)	Aluminum metal matrix	OM-47	PV/T	PV-temperature reduction improved by 11.1% Maximum thermal efficiency was 48.5% Electrical efficiency enhanced by 1.3%
Maiti et al. (Maiti et al., 2011)	Aluminum chips	Paraffin	PV integrated with V-trough	Melting time of PCM was shortened by 70 min PV temperature dropped by a maximum of 22 °C 55% overall output power improvement
Rad et al. (Vaziri Rad et al., 2021)	Aluminum shavings	Salt hydrate	PV/T	Melting time of PCM was shortened by 19–25% PV temperature declined by 20 °C Average thermal efficiency was 60.9% Electrical efficiency enhancement was 2.6% Average exergy efficiency was 17.4%
Klemm et al. (Klemm et al., 2017)	Metal fibers	RT54 HC	PV	PV temperature dropped by a maximum of 20 °C
Ahmadi et al. (Ahmadi et al., 2021)	PS-CNT foam	Paraffin wax	PV/T	The PV cell temperature dropped by 6.9% Electrical efficiency enhanced by 13.9%
Luo et al. (Luo et al., 2017)	Expandable graphite	RT28	PV	PV cell temperature declined by 25 °C Maximum improvement in power output was 11.4%

## Challenges and future directions

The enhancement of PV systems, particularly via cooling methods, is still in its infancy and have many challenges that need intensive investigations. The following are some recommended future works in the field of PV cooling:

- More evaluations and optimizations of effective cooling on large-scale grid-connected systems are required.
- Silicon is the best semiconductor material used in the manufacture of photovoltaic cells. But the search continues to find another material better than silicon that does not suffer from a lack of voltage when its temperature rises.
- It is necessary to search for a coating material used to paint the photovoltaic cells to cool them without affecting the amount of solar rays falling on them.
- An actual continuous thermal system (PV/T) with zero power required should be established based on a real application such as a desalination plant and HVAC.
- Analyses based on PV systems’ economic, environmental, energy, and exergy (4E) should be carried.
- Establishing stations that connect solar energy and wind energy is necessary to provide power throughout the morning and evening.
- Advanced experimentations are still required for evaluating the feasibility of organic and combined PCMs in PV cooling.
- For the PCM-based thermal management approach, it is suggested to use different kinds of PCMs with various melting points and thermophysical features to find the most favorable ones with highest thermal capacity and affordability.

## Conclusions

The working temperature of the photovoltaic cells is an important parameter that affects the performance of the PV cells, so the PV cells should be cooled to improve their performance. This research represents a comprehensive review of the different cooling techniques used in PV cooling, such as active cooling, passive cooling, PCM cooling, and PCM with additives. The main conclusions are as follows:

- The passive cooling technique is easy, simple, and low cost but is characterized by the low heat transfer rate, so it did not provide a high enhancement in the photovoltaic performance.
- The active cooling technique is considered an effective way to improve the photovoltaic performance, but it depends on an external power source, so the external power is deducted from the power produced from the PV cells, reducing the net output power produced from the PV cells.
- PCM cooling is considered one of the successful future methods in photovoltaic cooling, but due to the low thermal conductivity of the PCM, this method gives a low enhancement. The thermal conductivity of the PCM should be improved by adding another component to improve the cooling performance.
- Adding fins to the PCM is a rather primitive method and works to improve only the PCM adjacent to the fins without looking at the rest of the quantity.
- Adding the nanoparticles to the PCM is an innovative way to improve the performance of the PCM better than adding the fins and gives better results, but due to the high price of the nanoparticles, this method is considered uneconomic.
- Adding the porous metal to the PCM is an ideal way to improve the performance of PCM in cooling, and it is considered an economical method compared to adding nanoparticles.

## Data Availability

Research data can be obtained from the corresponding author through email.
